# Durable Ru Nanocrystal with HfO_2_ Modification for Acidic Overall Water Splitting

**DOI:** 10.1007/s40820-024-01384-7

**Published:** 2024-04-30

**Authors:** Xiangkai Kong, Jie Xu, Zhicheng Ju, Changle Chen

**Affiliations:** 1 School of Materials and Physics, China University of Mining and Technology, Xuzhou, 221116 Jiangsu People’s Republic of China; 2 Anhui Province Key Laboratory of Pollutant Sensitive Materials and Environmental Remediation, Huaibei Normal University, Huaibei, 235000 Anhui People’s Republic of China; 3 School of Chemistry and Materials Science, University of Science and Technology of China, Hefei, 230026 Anhui People’s Republic of China; 4 School of Chemistry and Chemical Engineering, Nanjing University, Nanjing, 210023 Jiangsu People’s Republic of China

**Keywords:** Ruthenium, Hafnium dioxide, Oxygen evolution catalysis, Anti-oxidation

## Abstract

Durable and efficient bi-functional catalyst, that is capable of both oxygen evolution reaction and hydrogen evolution reaction under acidic condition, are highly desired for the commercialization of proton exchange membrane water electrolysis. Herein, we report a robust L-Ru/HfO_2_ heterostructure constructed via confining crystalline Ru nanodomains by HfO_2_ matrix. When assembled with a proton exchange membrane, the bi-functional L-Ru/HfO_2_ catalyst-based electrolyzer presents a voltage of 1.57 and 1.67 V to reach 100 and 300 mA cm^-2^ current density, prevailing most of previously reported Ru-based materials as well as commercial Pt/C||RuO_2_ electrolyzer. It is revealed that the synergistic effect of HfO_2_ modification and small crystalline domain formation significantly alleviates the over-oxidation of Ru. More importantly, this synergistic effect facilitates a dual-site oxide path during the oxygen evolution procedure via optimization of the binding configurations of oxygenated adsorbates. As a result, the Ru active sites maintain the metallic state along with reduced energy barrier for the rate-determining step (*O→*OOH). Both of water adsorption and dissociation (Volmer step) are strengthened, while a moderate hydrogen binding is achieved to accelerate the hydrogen desorption procedure (Tafel step). Consequently, the activity and stability of acidic overall water splitting are simultaneously enhanced.

**Supplementary Information:**

The online version contains supplementary material available at 10.1007/s40820-024-01384-7.

## Highlights

- Heterostructure constructed via confining crystalline ruthenium nanodomains by hafnium dioxide matrix was fabricated through a two-step annealing method for overall water splitting.
- The synergistic effect of hafnium dioxide modification and small crystalline domain formation significantly alleviates the over-oxidation of ruthenium.

**Supplementary Information:**

The online version contains supplementary material available at 10.1007/s40820-024-01384-7.

## Graphical abstract

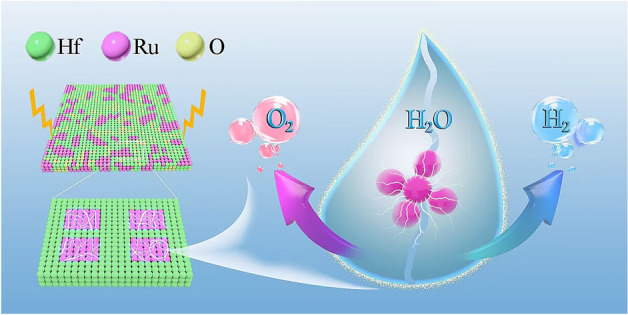

## Introduction

H_2_ production through electrochemical water splitting is considered as a promising and convenient approach to store intermittent renewable energy, with high combustion enthalpy and minimal CO_2_ footprint [1–6]. The past decades have witnessed growing research efforts on developing efficient electrocatalysts for its two half reactions, involving the anodic oxygen evolution reaction (OER) and cathodic hydrogen evolution reaction (HER) [7–11]. However, the reported OER catalysts normally work effectively in alkaline environments, while acidic conditions are more suitable for HER operation [12–14]. Such a pH mismatch for these two branch reactions severely hinders the electrolyzer to achieve industrial H_2_ production [15, 16]. Henceforth, the design and preparation of bi-functional electrocatalysts for overall water splitting under acidic condition are highly desired [17], but have been recognized as a long standing challenge.

Transition metal phosphides, hydroxides, phosphates and selenides have recently been demonstrated to be promising alkaline OER electrocatalysts, while their HER and acidic OER properties have remained largely unexplored [18–21]. Among these candidates, Ru-based nanomaterials demonstrate substantial potential for both OER and HER. On the one side, RuO_2_ is deemed as an ideal alternative to expedite the kinetics of oxygen production, which is commonly used as a benchmark to evaluate the performance of OER catalysts [22–24]. On the other side, Ru metal possesses a comparable affinity to hydrogen (≈65 kcal mol^-1^) as Pt (≈62 kcal mol^-1^), affording promising performance toward hydrogen generation [25–27]. Since Ru is much cheaper as compared with other precious metals, the development of bi-functional Ru-based catalysts for overall water splitting is expected to reduce the production cost and simultaneously simplify the practical operation process. Despite the good hydrogen production capability of metallic Ru, the over-oxidation of Ru during OER can create highly soluble Ru^*n*+^ species (*n* > 4), leading to quick degradation and significant loss in oxygen generation activity [28–32]. Accordingly, various strategies of elements decoration have been developed to improve the durability of Ru-based materials for OER. For example, elements including Mn [33], Cu [34], Ni [35], Pt [36] have been shown to modify the chemical environment of RuO_2_ via donating electrons into the RuO_2_ matrix, alleviating its undesired dissolution via over-oxidation. Thus, stabilizing low-valent Ru-based catalysts is necessary for achieving good stability during electro-oxidation operation. Unfortunately, the more stable low-valent RuO_2-*x*_ species generally deliver a slower kinetics during OER than high-valent Ru species [31, 37, 38]. Additionally, this electron-injection approach cannot simultaneously meet both hydrogen and oxygen production. Hence, a new strategy is required to address this dilemma and enable high-performance overall water splitting.

Recently, Kim and coworkers demonstrated that metallic Ru possesses a better activity for OER when compared with its oxides [39]. However, it is unstable under high applied potentials during OER operation, leading to gradual dissolution via oxidation into a higher Ru^*n*+^ (*n*>4) state. Therefore, the construction of robust Ru nanocrystals holds great potentials to simultaneously achieve sustained oxygen production as well as ideal hydrogen generation. In this work, we hope to address this issue by incorporation of a guest material with strong oxidation resistance to prevent the metallic Ru from over-oxidation. Hf possesses an electron structure of 5*d*^2^6*s*^2^, enabling the highest valence state of Hf in its dioxide compound. HfO_2_ has attracted considerable interest in thermal catalysis due to its high resistance to corrosion and oxidation [40, 41]. It mostly takes a supporting role because of the moderately lower bandgap when compared with traditional substrates such as SiO_2_ or Al_2_O_3_ [42]. Recent studies reveal that the synergistic effect of Ru and Hf can improve the hydrogen evolution [25, 43]. Herein, Ru nanocrystals with small crystalline domains were created and HfO_2_ was introduced to protect Ru against over-oxidation. The electrochemical measurements suggested the as-obtained catalyst required a low overpotential of 197 mV to reach 10 mA cm^-2^ for acidic OER, accompanied by a stable behavior over 250 h operation. Meanwhile, the HER performance was also enhanced, which enabled a stable and efficient electrocatalyst for acidic overall water splitting.

## Experimental Section

### Materials

Ruthenium acetylacetonate (97%), hafnium(IV) acetylacetonate (97%) and potassium bromide (99%) were purchased from Shanghai Macklin Biochemical Co., Ltd. All chemicals were used as received without further purification. Deionized water was used in all the experiments.

### Synthesis

S-Ru-HfO_2_ was prepared by a facile solid-phase reaction method. In a typical synthesis, 40 mg of ruthenium acetylacetonate, 20 mg of hafnium acetylacetonate, and 120 mg of potassium bromide were mixed in a solution composed of 10 mL of deionized water and 20 mL of absolute ethanol under vigorous sonication and stirring. After drying overnight in an oven at 60 °C, the obtained powder mixture was transferred into a tube furnace. The reactor was calcined at 290 °C with a heating rate of 5 °C min^-1^ and then, maintained at that temperature for 2 h under air condition. When the reaction was completed, this system was naturally cooled to the room temperature. Afterward, the product was washed several times with alcohol and water to remove the introduced KBr and then, freeze-dried. The obtained catalyst was denoted as S-Ru/HfO_2_, with “S” representing the short time calcination. After that, the sample was further annealed in nitrogen atmosphere at 290 °C for another 10 h, and the final product was labeled as L-Ru/HfO_2_, meaning a long-time annealing processing.

An amount of S-Ru-HfO_2_ was further annealed at 800 °C in N_2_ for 10 h, with the same heating rate of 5 °C min^-1^, and the obtained sample was referred as H-Ru/HfO_2_. For comparison, we also prepared the corresponding pure Ru sample in the same way as above except without hafnium acetylacetonate, and the achieved samples were labeled as S-Ru and L-Ru, respectively.

### Characterizations

Powder X-ray diffraction (XRD) was carried out on Panalytical Empyrean with Cu Kα radiation for the crystalline phase analysis. The structures of the prepared samples were characterized by transmission electron microscopy (TEM) images (JEOL JEM-2100F) and scanning electron microscopy (SEM) images (HITACHI REGULUS-8220). X-ray photoelectron spectroscopy (Kratos Axis Supra+) was adopted to perform elemental analysis and determine the composition of the materials. The Raman spectra were recorded by the confocal laser microscopic Raman spectrometer (viaReflex). Fourier transform infrared (FTIR) spectra were recorded using a Nicolet iS50 Spectrophotometer (Thermo-Scientific). The X-ray absorption fine structure spectra (XAFS) were collected with Si(111) crystal monochromators at BL11B beamlines in the Shanghai Synchrotron Radiation Facility (SSRF) (Shanghai, China). In situ Raman spectroscopy was recorded on the XPLORA PLUS Raman spectrometer. The electrochemical cells used for Raman measurements are homemade from Teflon, with a quartz plate serving as a window through the laser. The synthesized sample was used as the working electrode and the opposite electrode, and the Ag/AgCl electrode (1.0 M KCl as the filled electrolyte) was used as the reference electrode. In order to apply a control potential to the catalyst during Raman measurements, chronoamperometry was performed at different potentials of 0–1.6 V in 0.5 M H_2_SO_4_. The inductively coupled plasma (ICP) measurement was conducted on iCAP7400(Thermo Fisher). The thickness information was characterized on an atomic force microscope (AFM; Multimode 8, Bruker) with tapping mode. Brunauer–Emmett–Teller (BET) measurement was carried out on ASAP 2460 Version 3.01.

### Electrochemical Measurements

All of the electrochemical measurements were performed on the electrochemical workstation (CHI 760e, CH Instruments, Inc.), the Ag/AgCl electrode electrode was used as the reference electrode, the graphite rod was used as the counter electrode, and the as-synthesized samples were used as the working electrode to study the electrocatalytic performance. 5 mg of catalyst and 20 μL of Nafion solution (Aldrich, 5 wt%) were dispersed in 2.5 mL of water-isopropanol solution at a volume ratio of 3:2 and sonicated for 20 min to form a homogeneous ink. Load 5 μL of catalyst ink (containing 10 μg of catalyst) on a polished glassy carbon electrode with a diameter of 3 mm. A 0.5 M H_2_SO_4_ aqueous solution was selected as the electrolyte and purged with pure O_2_ and H_2_ for OER and HER measurement, respectively. The linear sweep potential with a sweep rate of 5 mV s^−1^ was calibrated as a reversible hydrogen electrode (RHE). The Nyquist plot is done in the range of 100 kHz to 0.05 Hz, with an amplitude of 5 mV and an overpotential of 50 mV. In addition, 1.0 M PBS solution and 1.0 M KOH were used to evaluate their performance in the neutral and alkaline electrolytes. The value of turnover frequency (TOF) is calculated by assuming that every metal atom is involved in catalysis (the TOF lower limit): TOF = *J*/(4×*F*×*n*), where *J* is at 50 mV, and the number 4 represents per mole O_2_ has 4 electrons, *F* is the Faraday constant (96,485.3 C mol^-1^), and *n* is the number of moles of metal atoms evaluated by ICP measurement.

### Density Functional Theory Calculations

The density functional theory (DFT) calculation was performed on the VASP 5.3 software. The PBE-type GGA functional was used to treat the exchange-correlation energy, and the projector augmented wave (PAW) technique was used for the ion-electron interaction. During the geometric optimization, both lattice constants and atomic positions were relaxed. The convergence criterion was set as forces on each atom less than 0.02 eV Å^-1^ and total energy change smaller than 1.0 × 10^-5^ eV. The DFT-D3 method was employed for the Van der Waals force correction. The adsorption energy was calculated as the difference between the energies for the adsorbed structure and their free or isolated models.

## Results and Discussion

### Synthesis and Characterizations of Ru/HfO_2_ Electrocatalysts

**Fig. 1:**
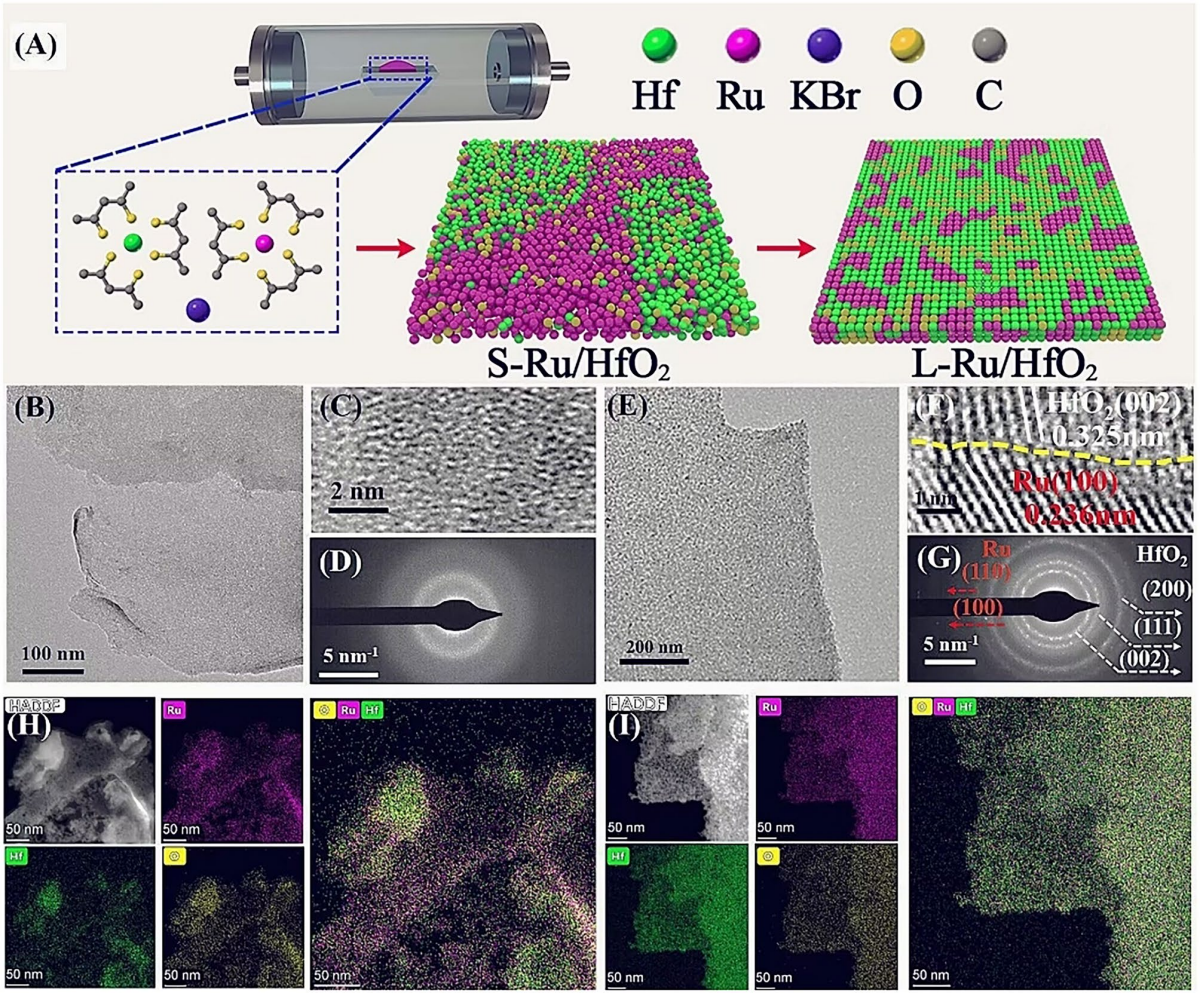
**A** Schematic illustration for the synthesis of L-Ru/HfO_2_ catalyst. Characterization of S-Ru/HfO_2_: **B** TEM and **C** HRTEM images, **D** SAED pattern. Characterization of L-Ru/HfO_2_: **E** TEM and **F** HRTEM images, **G** SAED pattern. HAADF-STEM images and corresponding EDX elemental mapping for **H** S-Ru/HfO_2_ and **i** S-Ru/HfO_2_

The sample fabrication was conducted via a solid-state method as modified from literature, which has also been used in our recent studies to develop high-performance catalysts [44–46]. As illustrated in Fig. 1A, a certain amount of Ru(acac)_3_ and Hf(acac)_4_ was mixed with KBr for annealing, to achieve the hybrid of Ru and Hf in the composite, referred as S-Ru/HfO_2_. In this system, KBr regulated the diffusion of metal atoms, leading to a sheet-like structure with low aggregation [47]. The slow diffusion rates of Ru and Hf on KBr surface could impair their combination, resulting in a weak interaction between themselves associated with a poor stability [45, 47]. Hence, we envisioned that an additional annealing after KBr removing may strengthen the interaction between these two components, which could not only regulate the chemical environment and structure of active sties, but also provide an improved durability via strengthening the binding within the sample system [44]. Subsequently, the KBr template was removed via carefully washing, followed by an additional annealing to promote the crystallinity, affording sample denoted as L-Ru/HfO_2_.

Figures S1 and 1B displayed the typical SEM and TEM images of S-Ru/HfO_2_, where a clear two-dimensional feature without any particle nor fragment was discerned over the whole images, revealing the well flat configuration control during sample growth using this molten salt method. The microstructure was further studied by high-resolution TEM (HRTEM) in Fig. 1C. Clearly, S-Ru/HfO_2_ exhibited a fuzzy pattern without any ordered atomic arrangement in the recorded image. In addition, diffused rings were observed in the selected area electron diffraction (SAED) pattern (Fig. 1D). These results indicated the amorphous structure of S-Ru/HfO_2_, which should be generated by the slow diffusion of Ru and Hf atoms on KBr template as observed in our previous studies [44, 45]. Although materials in amorphous phase normally exhibited high kinetics for catalysis [48], a rapid loss in activity was obtained owing to the existence of rich unsaturated atoms, lattice distortions as well as vacancies. This feature led to a low stability and thus severely hindered its practical application. In order to enhance the contact between Ru and Hf species, a subsequent thermal annealing was performed accordingly. Both SEM (Fig. S2) and TEM (Fig. 1E) images demonstrated that the sheet-like morphology was well maintained. The AFM image indicated a height profile of approximately 4.8 nm in thickness (Fig. S3). Of note, roughing occurred on the surface of L-Ru/HfO_2_. Brunauer–Emmett–Teller (BET) measurement indicated an increased surface area from 46.61 to 56.00 m^2^ g^-1^ for S-Ru/HfO_2_ and L-Ru/HfO_2_, respectively, and both of them were assigned to type IV isotherms [49] (Fig. S4). Apparently, crystallographic fringes appeared in the recorded HRTEM image (Fig. 1F). The lattice spacing of 0.325 and 0.236 nm was assigned to orthogonal HfO_2_ (002) (PDF#40-1173) and hexagonal Ru (100) (PDF#89-3942) crystallographic planes, respectively. Clear interface was discerned between Ru and HfO_2_, which suggested the intimate contact formed at their interface region. Meanwhile, the diffractive rings appeared in Fig. 1G confirmed the improved crystallinity of Ru and HfO_2_ in L-Ru/HfO_2_. All of these comparisons illustrated the effective modification on microstructure of the hetero-interface by post-annealing.

**Fig. 2:**
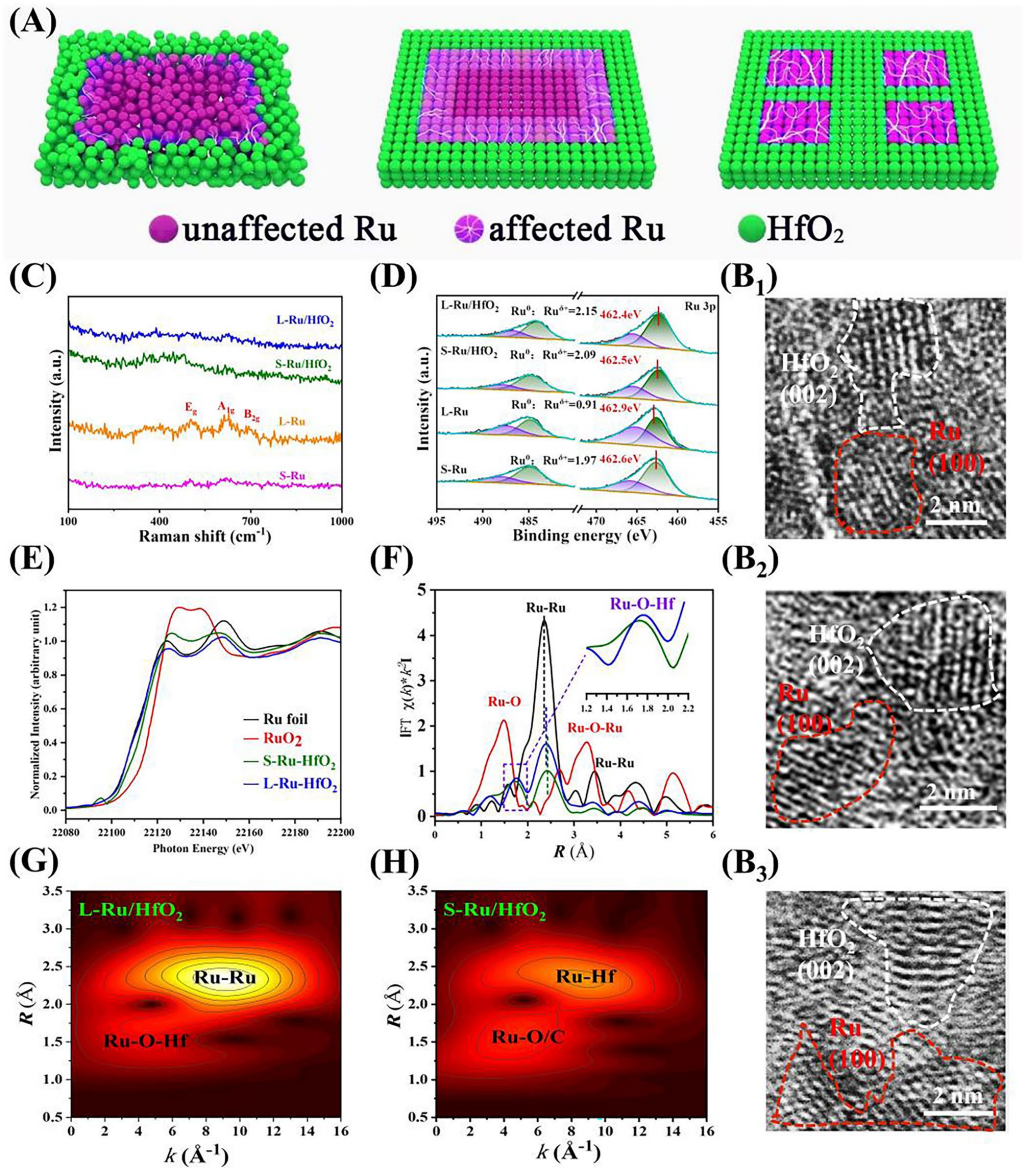
**A** Schematic illustration of the interaction between Ru and HfO_2_. **B** HRTEM images for L-Ru/HfO_2_. Physical characterizations on these samples: **C** Raman spectra, **D** High resolution XPS spectra of Ru 3*p* orbital, **E** XANES spectra, **F** Ru K-edge EXAFS spectra, and WT-EXAFS spectra for **G** L-Ru/HfO_2_ and **H** S-Ru/HfO_2_

An intimate interfacial interaction could ensure improved structure stability for both Ru and HfO_2_, which held great potential to achieve high durability during operation. As depicted in the left of Fig. 2A, the disordered arrangement in amorphous materials would result in weak interactions between the atoms of both same type and different species, thereby leading to a weak Ru-HfO_2_ contact. Controlled annealing was able to increase the crystallinity of both Ru and HfO_2_, offering an ordered atomic arrangement and enhancing their interfacial interaction. However, this interface effect was closely related to the size of crystalline domains. For a large-sized Ru crystal, the interior Ru atoms almost remained as before, only edged Ru atoms around the interface were affected (middle in Fig. 2A). In contrast, when the crystalline Ru domain significantly decreased, most Ru atoms could be protected by their surrounding HfO_2_ layer, enabling a higher resistance toward oxidation and corrosion (right panel in Fig. 2A). Henceforth, constructing heterostructure with small crystalline regions would benefit the interaction between Ru and HfO_2_. HRTEM characterizations demonstrated that all of the generated Ru and HfO_2_ crystalline domains were less than 5 nm (Fig. 2B). This was consistent with the XRD observation as no diffractive peaks existing on the recorded pattern of L-Ru/HfO_2_ (Fig. S5). Furthermore, high-angle annular dark-field scanning TEM (HAADF-STEM) image and the corresponding energy-dispersive X-ray spectroscopy (STEM-EDS) elemental mapping were performed (Fig. 1H, I). Obviously, S-Ru/HfO_2_ displayed a discrete distribution for Ru and Hf over the sheet-like catalyst, while their distribution became quite uniform for L-Ru/HfO_2_. This further suggested the post-annealing drove the diffusion of atoms to make a more intimate interaction between these two components.

Raman spectra were subsequently performed to give more insights on the interface structure between Ru and HfO_2_ (Fig. 2C). There are three peaks located at 491.7, 621.8, and 681.5 cm^-1^ on pure Ru nanosheets (both of L-Ru and S-Ru), which corresponded to E_g_, A_1g_, and B_2g_ vibration modes of Ru-O bond, respectively [50]. The intensity of these peaks became much stronger for L-Ru than those of S-Ru, indicating the high sensitivity of bare Ru to oxygen, which may result from the partial oxidation of Ru when exposed to air [51]. Interestingly, these vibration peaks almost disappeared on S-Ru/HfO_2_ and L-Ru/HfO_2_, suggesting the HfO_2_ incorporation could effectively prevent Ru metal from oxidation. After that, X-ray diffraction photoelectron spectroscopy (XPS) was conducted to detect their surface chemical state and elemental composition. The recorded survey spectrum of L-Ru/HfO_2_ showed the presence of Ru, Hf, C, and O elements in the catalyst (Fig. S6). The peaks at 484.4 and 462.3 eV in the high-resolution XPS profile corresponded to Ru 3*p*_1/2_ and Ru 3*p*_3/2_, respectively (Fig. 2D). The prominent Ru 3*p*_3/2_ peak exhibited a positive shift of 0.3 eV for L-Ru as compared with that of L-Ru/HfO_2_. Furthermore, it was deconvolved to Ru^0^ (462.1 eV) and Ru^δ+^ (465.4 eV) [44]. The ratio of Ru^0^/Ru^δ+^ for S-Ru was 1.97, which significantly decreased to 0.91 after the post-annealing (L-Ru). In contrast, this ratio showed a slight change from S-Ru/HfO_2_ (2.09) to L-Ru/HfO_2_ (2.15). Additionally, the amount of Ru^0^ in the heterostructure catalyst was much greater than that of pure Ru without HfO_2_ protection. The comparison of O 1*s* spectra also suggested more Ru^0^ content in L-Ru/HfO_2_, with a smaller portion of lattice O as compared with L-Ru in Fig. S7. These results validated the HfO_2_ moderation endowed Ru metal with a higher resistance toward oxidation, which held the potential to provide improved stability for Ru in OER operation as well as a high HER response to realize overall water splitting. In the meantime, Fig. S8 addressed the positive shift of Hf 4*f* signal for L-Ru/HfO_2_ than L-Hf, unveiling electrons transferring from HfO_2_ to Ru. This benefited to maintain Ru site in the metallic state during oxidation.

Afterward, X-ray absorption spectroscopy (XAS) was conducted to investigate the electronic configuration and local chemical environment of L-Ru/HfO_2_. As shown in Fig. 2E, X-ray absorption near-edge structure (XANES) analysis demonstrated that the Ru K-edge of S-Ru/HfO_2_ suited between commercial Ru foil and RuO_2_, which suggested the metaphase of Ru with partial positively charged state between Ru^0^ and Ru^4+^ [50]. Notably, L-Ru/HfO_2_ exhibited a much more negative absorption edge than S-Ru/HfO_2_, which almost coincided with that of Ru foil. Beyond, the white line intensity of Ru foil was smaller than S-Ru/HfO_2_ but larger than L-Ru/HfO_2_, revealing the majority of Ru atoms in L-Ru/HfO_2_ possessed Ru^0^ state with negligible oxidation. Furthermore, the peak of S-Ru/HfO_2_ in the pre-edge region disappeared on L-Ru/HfO_2_, which indicated the strengthened interaction between Ru and HfO_2_, with more empty orbitals of Ru being filled by the electron injection during the post-annealing procedure. The two scattering peaks observed at ~2.4 and 1.7 Å in EXAFS were indexed to Ru-Ru and Ru-O-Hf coordination, respectively (Fig. 2F and Table S1). Apparently, the Ru-Ru peak of L-Ru/HfO_2_ was significantly stronger than Ru-O, verifying the majority of metallic Ru state in the crystalline heterostructure. In contrast, S-Ru/HfO_2_ exhibited a relatively high intensity for Ru-O peak but the intensity of Ru-Ru bonding was much lower as compared. The enlarged view in Fig. 2F unraveled the Ru-O-Hf peak of L-Ru/HfO_2_ shifted to a smaller distance when compared to that of S-Ru/HfO_2_, presumably originating from the stronger interaction imposed between Ru and HfO_2_. The Ru-Ru coordination numbers increased from 4.7 to 6.6 as S-Ru/HfO_2_ transformed to L-Ru/HfO_2_ (Table S1), further confirming the higher crystallinity induced more Ru atoms to be coordinated and protected by HfO_2_. On basis of these discussions, it was concluded that the Ru sites were more effectively protected by the HfO_2_ matrix in L-Ru/HfO_2_ and thereby generated a higher oxidation resistance for the higher crystalline catalyst during operation. Besides, the wavelet transforms of Ru-edge EXAFS oscillations demonstrated the maximum-intensity values of L-Ru/HfO_2_ were located at *k* ≈ 4.9 and 9.1 Å^−1^, which were assigned to Ru-O-Hf and Ru-Ru scattering paths, respectively (Fig. 2G). It also reflected that L-Ru/HfO_2_ possessed more metallic Ru states and fewer Ru-O bonds than S-Ru/HfO_2_ (Fig. 2G, H), exhibiting a metallic feature that quite close to Ru foil. Since ICP measurements disclosed a similar atomic ratio of Ru to Hf for S-Ru/HfO_2_ (0.88) and L-Ru/HfO_2_ (0.80), we attributed the more stable Ru^0^ state of L-Ru/HfO_2_ to the enhanced interfacial interaction constructed by increasing the crystallinity of Ru and HfO_2_.

As a control, S-Ru/HfO_2_ was annealed at 800 °C to increase the crystalline domain size for comparison, and the obtained sample was denoted as H-Ru/HfO_2_. Obviously, strong diffraction peaks were found in the recorded XRD pattern, which were well assigned to hexagonal Ru and HfO_2_ as marked in Fig. S9. These intensified diffraction peaks reflected the further growth of crystalline domains in the sample. The main Ru peak became rather sharper and stronger, implying large-size crystalline Ru domains were formed as expected.

### Electrocatalytic Performances

**Fig. 3:**
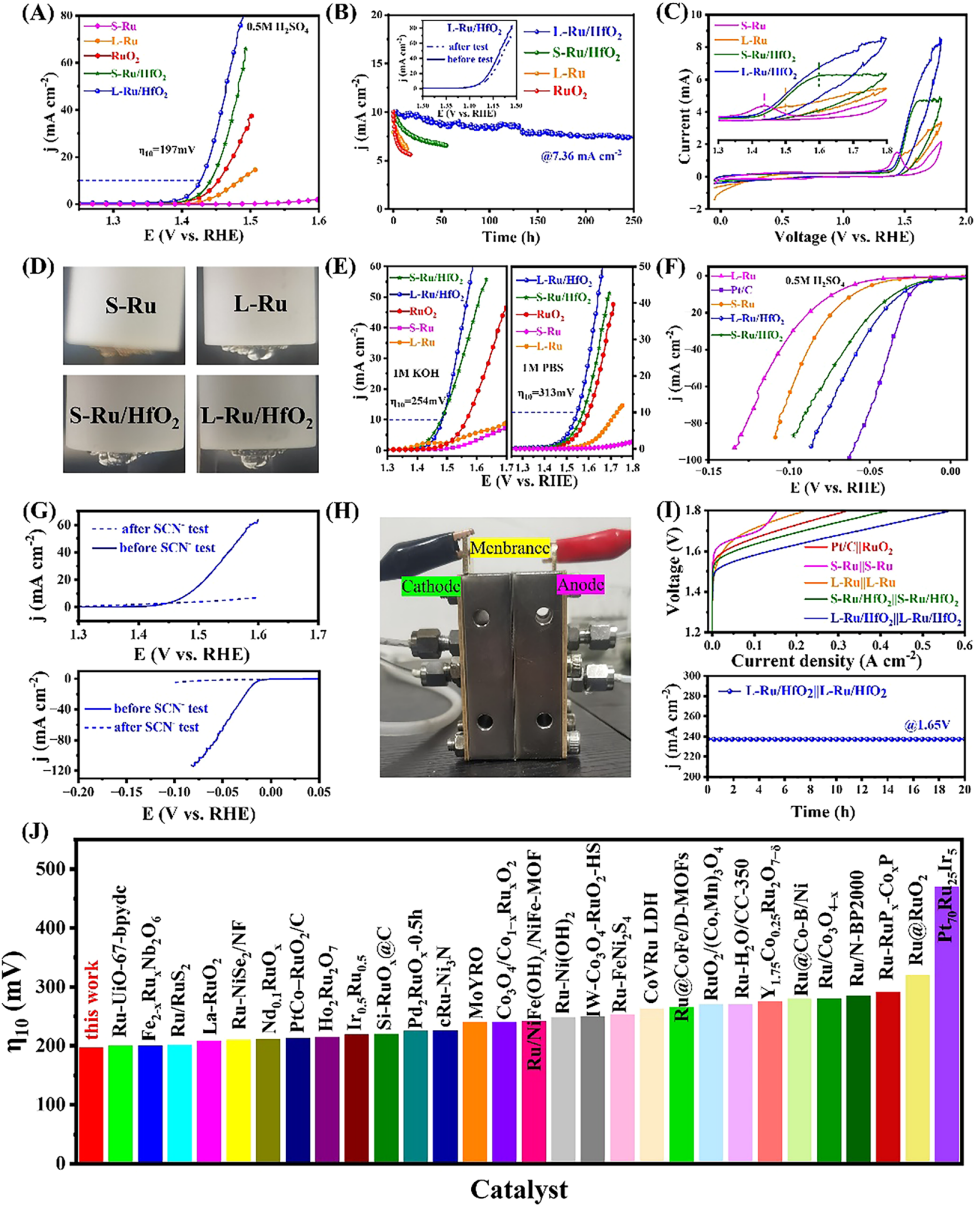
The electrochemical measurements of these samples in the three-electrode system in 0.5 M H_2_SO_4_ solution: **A** LSV curves, **B** the chronoamperometry measurement with the *i–t* response at 10 mA cm^-2^, and the LSV polarization curves recorded before and after were inset in, and **C** the first cycle of CV scan during OER activation. **D** The digital photos observed on electrode surface during the first cycle of CV scan for each sample activation. **E** OER LSV curves for these sampled measured in 1.0 M KOH and 1.0 M PBS. **F** HER LSV curves for these sampled measured in 0.5 M H_2_SO_4_ solution. **G** Comparison of LSV curves obtained during OER and HER operation in the KSCN involved electrolyte. **H** The digital image of the assembled PEM electrolyzer. **I** Polarization curves achieved on these catalysts assembled PEM electrolyzers and durability estimation on L-Ru/HfO_2_||L-Ru/HfO_2_ coupled PEM electrolyzer. **J** Comparison of η_10_ for acidic OER on L-Ru/HfO_2_ with recently reported Ru-based catalysts. The detailed information for these compared catalysts can be found in the supporting information file.

Electrochemical measurement was subsequently carried out to evaluate the water splitting performance using a three-electrode technique. First, we examined their OER behavior in O_2_-saturated 0.5 M H_2_SO_4_ because of its sluggish kinetics as compared with HER. Figure 3A illustrated both S-Ru/HfO_2_ and L-Ru/HfO_2_ displayed a more negative potential than the commercial RuO_2_ reference, at which the recorded current density increased sharply. Specifically, L-Ru/HfO_2_ exhibited the smallest onset potential among these catalysts, with their order following: L-Ru/HfO_2_ (1.38 V) < S-Ru/HfO_2_ (1.40 V) < RuO_2_ (1.42 V) < L-Ru (1.43 V) < S-Ru (1.53 V). Meantime, the required overpotential for L-Ru/HfO_2_ to achieve the benchmark 10 mA cm^-2^ current density was 197 mV, which was lower than S-Ru/HfO_2_ (212 mV) and much smaller than those of RuO_2_ (234 mV), L-Ru (256 mV) and S-Ru (>400 mV). Figure S10 addressed the optimization of Ru metal content, which indicated that 40 mg Ru(acac)_3_ is the optimal precursor dosage. Moreover, the smallest Tafel slope of L-Ru/HfO_2_ (46.8 mV dec^-1^) in Fig. S11 signified the much faster oxygen generation kinetics acquired on the catalyst with small crystalline domains. Accordingly, the electrochemical impedance spectroscopy (EIS) was measured at an overpotential of 200 mV, and the obtained curves confirmed the accelerated oxygen production on L-Ru/HfO_2_ (Fig. S12). Also, L-Ru/HfO_2_ and S-Ru/HfO_2_ overperformed H-Ru/HfO_2_ for OER operation (Fig. S13). Furthermore, both the double layer capacitance and turnover frequency imposed the advance of L-Ru/HfO_2_ (Fig. S14). As a result, L-Ru/HfO_2_ demonstrated excellent OER activity which exceeded most recently reported RuO_2_-based acidic OER catalysts (Fig. 3J).

Besides, the catalytic durability of L-Ru/HfO_2_ was examined using a chronoamperometry approach, through keeping the potential corresponding to the current density of 10 mA cm^-2^. The recorded i-t profiles were collected in Fig. 3B. Overall, L-Ru/HfO_2_ exhibited superior stability compared to other samples. Specifically, the initial current density of L-Ru/HfO_2_ decreased to 7.36 mA cm^-2^ after 250 h of acidic oxygen production, representing 73% current density was well retained during the long-term operation. Instead, other samples demonstrated rapid decays within 50 h. For instance, S-Ru underwent severe oxidation to form soluble high-valent Ru oxides, so its activity disappeared just within hundreds of seconds. The measured polarization curves demonstrated slight difference between linear sweep voltammetry (LSV) before and after the continuous OER evaluation for L-Ru/HfO_2_ (inset in Fig. 3B). What’s more, the first cyclic voltammetry (CV) cycle of each sample during catalyst activation was collected for comparison. As shown in Fig. 3C, pure Ru catalysts displayed an apparent oxidized peak around 1.47 V, which was assigned to continuous oxidation of RuO_2_ through a transition state to Ru(V) or Ru(VI) driven by applied oxidized potentials [52]. When combined with HfO_2_, this oxidation peak exhibited an obvious positive shift to 1.60 V for S-Ru/HfO_2_. Furthermore, this peak became rather featureless on L-Ru/HfO_2_, confirming the strengthened antioxidant effects on Ru endowed by crystalline HfO_2_ protection. This behavior was in agreement with the image as observed on the electrode surface in Fig. 3D. Clearly, the electrolyte around S-Ru electrode appeared yellow color, which was attributed to the rapid dissolution of over-oxidized Ru-based species. Likely, L-Ru/HfO_2_ exhibited colorless and transparent as the original electrolyte did, accompanied by large bubbles generated on the electrode surface. Therefore, the HfO_2_ presence afforded an improved anti-oxidation for L-Ru/HfO_2_ to keep Ru in metallic state as expected. Besides, the structure characterization indicated L-Ru/HfO_2_ could maintain the original sheet-like morphology with fine crystalline domains of Ru and HfO_2_ after subjecting the long-term stability estimation (Fig. S15). The ICP measurement suggested the atomic Ru/Hf ratio decreased to 0.68 after the durability evaluation. The obtained Ru 3*p*_3/2_ XPS spectra further verified that substantial Ru atoms maintained in metallic state under HfO_2_ protection. However, the atoms of L-Ru and S-Ru were almost transformed to RuO_2_ during OER operation (Fig. S16). These results illustrated a relatively small change in structure and composition of L-Ru/HfO_2_ during the 250 h of OER testing and also affirmed the stability enhancement as synergistically modified by HfO_2_ decoration and forming small crystalline domains.

A pH-universal catalyst was desired to adapt to different application scenarios. Accordingly, we studied the OER behavior of L-Ru/HfO_2_ in alkaline and neutral environment. As seen in Fig. 3E, L-Ru/HfO_2_ required an overpotential of 254 and 313 mV to reach 10 mA cm^-2^ in 1 M KOH and 1 M PBS electrolytes, respectively, which were better than all the controlled samples including RuO_2_. In addition, as the counterpart reaction for OER, hydrogen evolution performance of these as-synthesized samples was also investigated and compared with commercial Pt/C (Figs. 3F and S17). Obviously, L-Ru/HfO_2_ needed an overpotential of 18.9 mV to reach the reference current density of 10 mA cm^-2^ toward HER in 1 M KOH, overperformed other samples as well as commercial Pt/C catalyst (46.1 mV) (Fig. S17A). In the meantime, the onset potential of L-Ru/HfO_2_ (6.71 mV) was smaller than those of all controlled materials. Also, L-Ru/HfO_2_ exhibited the smallest Tafel slope (25.1 mV dec^−1^) among them, implying an accelerated Volmer-Tafel kinetic (Fig. S18A). The achieved small impedance of 15.1 Ω demonstrated a high charge transfer capacity for L-Ru/HfO_2_ (Fig. S18B). And the chronoamperometry test proved its good stability for continuous hydrogen production (Fig. S19). Moreover, the HER performance was examined in 1.0 M PBS, where L-Ru/HfO_2_ catalyst still exhibited the highest activity with 37.9 mV overpotential requirement to achieve 10 mA cm^-2^ current density in the neutral solution (Fig. S17B). In acidic environment, L-Ru/HfO_2_ overperformed all the Ru-based reference samples (Fig. 3F), implying the strengthened water dissociation ability of L-Ru/HfO_2_ to supply protons from water molecule for reduction. Since the OH* reactant also came from water dissociation for non-alkaline OER, this observation implied the Ru metal with HfO_2_ modification enabled an improved oxygen production in the acidic environment.

Next, control experiment was performed to disclose the pivotal role of Ru for this overall catalysis, by means of the blocking effect of SCN^-^ ions on Ru atoms [40]. As observed in Fig. 3G, L-Ru/HfO_2_ almost lost its catalytic ability for both OER and HER in presence of KSCN, with negligible current density responses. As such, HfO_2_ was inactive toward both OER and HER, and the actual Ru sites with HfO_2_ protection should determine the overall water splitting. Therefore, constructing Ru/HfO_2_ heterostructure with small crystalline domains could endow Ru sites with an enhanced stability, accompanied by an improved intrinsic activity. Finally, we assembled a proton exchange membrane (PEM) to study the potential of practical application. The as-achieved L-Ru/HfO_2_ was employed as both cathode and anode catalysts, which were further assembled with a Nafion 115 membrane in 0.5 M H_2_SO_4_ to construct an electrolyzer for overall water splitting evaluation (Fig. 3H). As compared in Fig. 3I, L-Ru/HfO_2_ needed 1.57 and 1.67 V voltages to reach the cell current densities of 100 and 300 mA cm^-2^, respectively, which were superior to S-Ru/HfO_2_ (1.63 V at 100 mA cm^−2^ and 1.74 V at 300 mA cm^−2^) and commercial Pt/C||RuO_2_ (1.66 V at 100 mA cm^-2^ and 1.79 V at 300 mA cm^-2^). The behavior was in consistent with the above observations as obtained by three-electrode measurement, confirming the efficient kinetics for this bi-functional catalyst. Apart from this, L-Ru/HfO_2_ assembled electrolyzer presented no significant attenuation of current density after a long period of water electrolysis (Fig. 3I), which was similar to above HER and OER stability measurements, demonstrating its potential for practical use in the water electrolyzer system.

### In Situ Spectra Characterizations and DFT Calculations

**Fig. 4:**
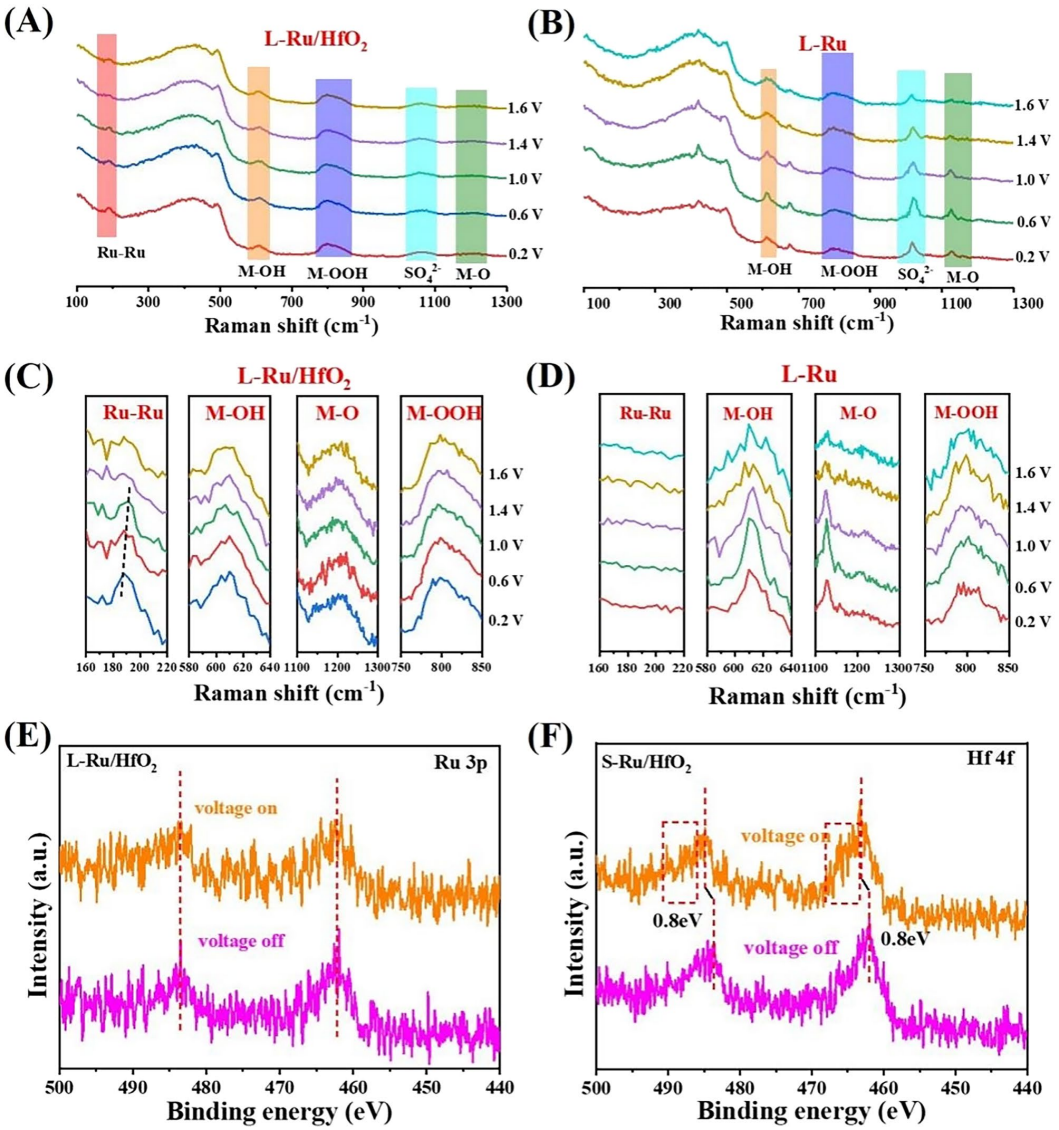
In situ Raman spectra recorded on **A** L-Ru/HfO_2_ and **B** L-Ru. **C, D** The magnified spectra of corresponding vibration regions for Fig. A and B, respectively. The in situ high-resolution XPS spectra of Ru 3*p* signals under an applied voltage of 2.0 V for **E** L-Ru/HfO_2_ and **F** S-Ru/HfO_2_

The electrochemical in situ Raman spectroscopy was then carried out to gain deeper insights on L-Ru/HfO_2_ for acidic oxygen evolution. As shown in Fig. 4A, the Raman peak at 187.9 cm^-1^ on L-Ru/HfO_2_ was assigned to the Ru-Ru binding vibration, while L-Ru without HfO_2_ modification displayed featureless on this signal (Fig. 4B). This verified that the majority of Ru atoms in L-Ru/HfO_2_ maintained metallic state, but the Ru atoms of L-Ru, especially those on surface, were preferable in an oxidized state. In a closer observation, the Ru-Ru peak of L-Ru/HfO_2_ exhibited a gradual shift toward the positive direction following potential increasing (Fig. 4C). When the applied potential was higher than 1.0 V, the Ru-Ru binding peak weakened and shifted negatively. Even up to 1.6 V, this bond signal was discerned in the recorded spectrum, suggesting the robust Ru^0^ state of L-Ru/HfO_2_ in the OER potential window region. Henceforth, HfO_2_ decoration significantly enhanced the anti-oxidation ability of Ru, thereby improving its stability. This was conductive to realize overall water splitting under a wide operating potential window. The vibration profiles at 609.8, 1198.2, and 798.8 cm^-1^ corresponded to M-OH [50], M-O [53] and M-OOH [54] binding modes, respectively, and the “M” represented active sites of catalyst for reaction (Fig. 4C, D). These oxygen related species were generally regarded as main intermediates evolved during oxygen production.

As compared with L-Ru, L-Ru/HfO_2_ exhibited a relatively steady M-OH profile, which was independent of potential variations. This indicated a stable chemical environment and robust structure for L-Ru/HfO_2_ to continuously supply OH from water molecule dissociation that could provide favorable reactants to ensure durable oxygen production. Previous studies reported the O*→OOH* conversion was the rate-determining step for OER, thus a moderate M-O bonding was beneficial to accelerate O_2_ production [39]. As seen in Fig. 4B, D, the M-O peaks were extremely sharp and strong on L-Ru. Accordingly, they became rather wider and weaker for L-Ru/HfO_2_ (Fig. 4A, C), indicating an effective alleviation to achieve moderate *O combination on Ru by HfO_2_ modification. This was conductive to reduce the energy barrier from *O to *OOH conversion, thereby ensuring the continuous progress of oxygen generation. Therefore, the M-OOH signals measured on L-Ru/HfO_2_ became quite stronger than those of L-Ru, which implied more *OOH species generated by HfO_2_ decoration due to its appropriate *O→*OOH conversion.

In order to examine the microelectronic structure response, in situ XPS characterization was thus performed. When applying a 2.0 V oxidized voltage, there was no significant change occurring in Ru 3*p* orbital of L-Ru/HfO_2_ (Figs. 4E and S20). However, it underwent a positive shift of 0.8 eV for S-Ru/HfO_2_ (Fig. 4F) as the external potential was applied. This represented that the crystalline improvement endowed Ru with a higher antioxidant capacity, which enabled it to maintain the metallic state under OER oxidation potentials and environment.

**Fig. 5:**
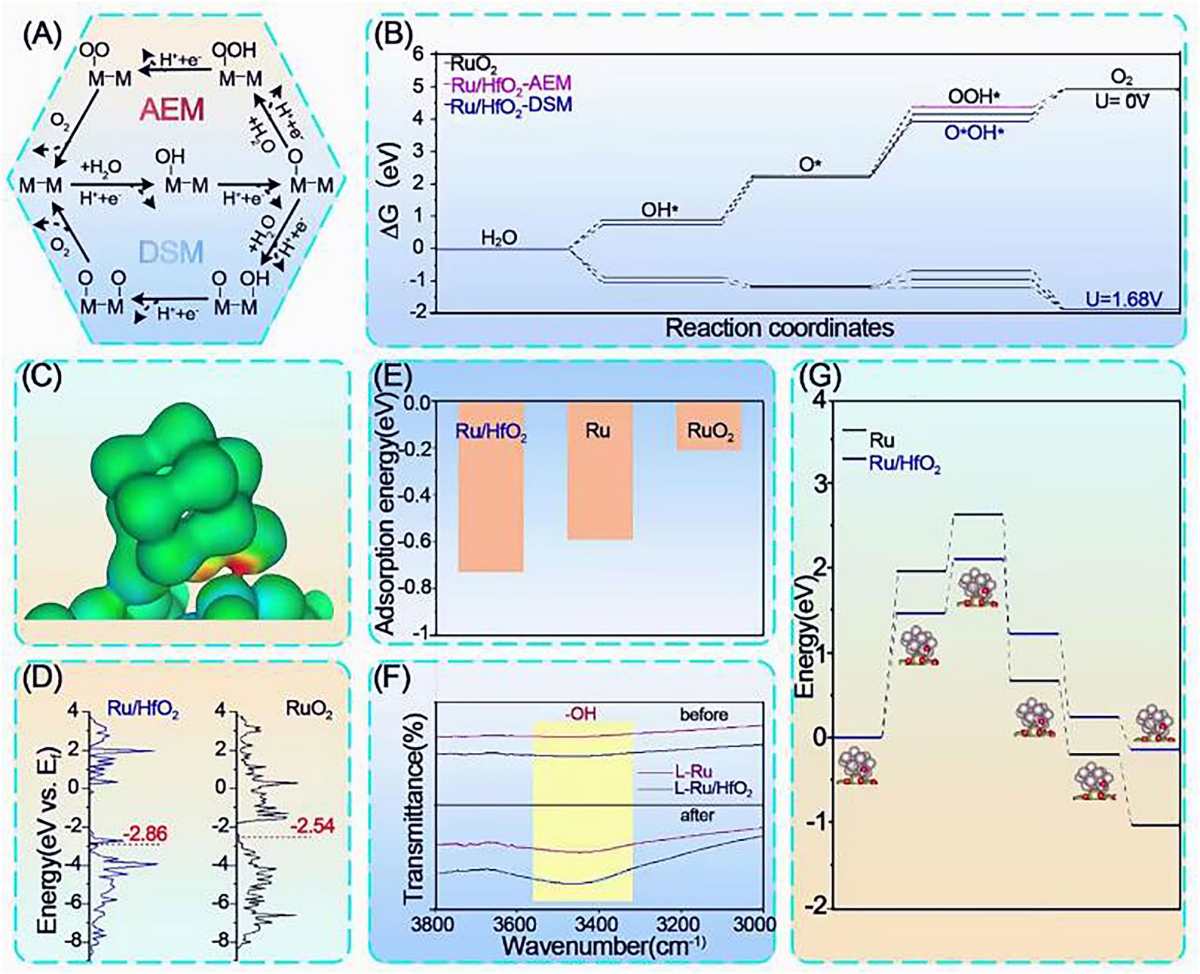
DFT calculations. **A** AEM and DSM paths of OER on the catalyst surface. **B** Free energy change diagrams for OER over Ru/HfO_2_ catalyst through different paths. **C** Charge density difference at the interface of Ru/HfO_2_, with the warm color and cold color representing charge accumulation and depletion, respectively. **D** Calculated projected DOS with *d*-band center highlighted for the active sites. **E** Adsorption energy of water molecule on these catalysts. **F** FTIR profiles of L-Ru and L-Ru/HfO_2_ before and after the water vapor treatment. **G** Water dissociation energy diagrams (inset: corresponding optimized Ru/HfO_2_ structures for water dissociation, with gray, light green, red and white balls representing Ru, Hf, O, and H atoms, respectively)

Subsequently, DFT calculations were performed according to experimental results. The HfO_2_ (001) crystal plane in orthorhombic symmetry and *Pmnb* space group was built to mimic HfO_2_ crystalline surface as observed in the TEM and XRD data. In light of previous reports, Ru13 cluster was selected to model the Ru counterpart [25]. Also, RuO_2_ (001) slab structure was taken and investigated for comparison. All of these structures were displayed in Fig. S21. Recent studies revealed three mechanisms for OER, including adsorbate evolution mechanism (AEM), lattice oxygen mechanism (LOM) and dual-site oxide mechanism (DSM) [25, 38]. Considering the Ru active sites in L-Ru/HfO_2_ were mainly kept in metallic state, the LOM pathway was excluded, and thus, we focused on the AEM and DSM in the following discussions, with their depiction shown in Fig. 5A. The corresponding free energy variation diagrams were compared in Fig. 5B, in which, each reaction step exhibited an endothermic feature at zero potential. Apparently, the conversion from O* to OOH* was the rate-determining step for Ru/HfO_2_, with a large barrier of 2.21 and 1.68 eV for the AEM and DSM paths, respectively, which were in agreement with previous reports. Although the limiting energy barrier of Ru/HfO_2_ in AEM pathway was greater than that of RuO_2_, its DSM root was significantly suppressed with a relatively lower barrier. Charge density difference calculation unveiled the electron transfer at interface, and the accumulated charge density at Ru sites was consistent with the XPS observations (Fig. 5C). The *d*-band center was sensitive to the intermediate adsorption. The presence of HfO_2_ enabled a downshift of *d*-band center position from −2.54 to −2.86 eV, making it farther away from the Fermi level as shown in the calculated density of states in Fig. 5D. Therefore, the interaction between adsorbed *O and active Ru site was weakened, facilitating the *O→*OOH conversion for continuous oxygen production.

Since H_2_O adsorption was the initial step that strongly affected the kinetics of acidic OER, we thereby evaluated its adsorption effect on Ru/HfO_2_. Apparently, Ru/HfO_2_ exhibited a significantly strengthened adsorption energy for H_2_O molecule than both pure Ru and RuO_2_ (Fig. 5E). This phenomenon was also confirmed by FTIR spectra. As compared in Fig. 5F, L-Ru/HfO_2_ exhibited an obvious increment in hydroxyl peak after being held in water vapor environment, while L-Ru displayed a less response to the water vapor adsorption. These results revealed the more feasible water adsorption ability on Ru benefited from HfO_2_ decoration, which facilitated water molecules to be continuously adsorbed on the catalyst surface in acidic environment, ensuring that the oxygen generation was kept running.

Furthermore, the transition state variation obtained from nudged elastic band calculations revealed a significantly suppressed energy barrier for Ru/HfO_2_ to dissociate water molecule into *OH and *H species (Fig. 5G). Thus, a strengthened water adsorption associated with an alleviated dissociation capability was simultaneously obtained by HfO_2_ modification, which enabled a boosted oxygen production rate on Ru/HfO_2_ in acidic media. On the other hand, *H adsorption free energy (ΔG*H) was considered as a key descriptor to describe the ability of adsorbed *H recombining H_2_ molecules and thus, was valid for predicting the HER capability. Figure S22 illustrated the quite thermoneutral ΔG*H on Ru/HfO_2_, facilitating H_2_ release and promoting the Tafel step of HER.

## Conclusions

In summary, L-Ru/HfO_2_ hybridized nanosheets composed of small crystalline domains were fabricated through a two-step annealing method, which could act as a stable catalyst for both acidic OER and HER, as well as in pH-universal conditions. The as-obtained L-Ru/HfO_2_ catalyst exhibited a low overpotential of 197 mV to reach 10 mA cm^-2^, associated with a slight degradation during continuous OER operation at 10 mA cm^-2^ for 250 h in 0.5 M H_2_SO_4_. When assembled with a proton exchange membrane, the bi-functional L-Ru/HfO_2_ catalyst-based electrolyzer presented a voltage of 1.57 and 1.67 V to reach 100 and 300 mA cm^-2^ current density, respectively, exceeding most reported Ru-based materials and commercial Pt/C||RuO_2_ electrolyzer. The electronic state study revealed the synergism of HfO_2_ modification and ultrasmall crystalline domain formation endowed Ru with improved anti-oxidation capability, leading to a metallic state that quite close to Ru foil. Both in situ Raman and in situ XPS characterizations confirmed the robust metallic Ru in L-Ru/HfO_2_ for catalysis. Combined with simulations, it suggested the limiting step of *O→*OOH conversion was accelerated through an dual-site oxide path and also the water adsorption and dissociation steps were strengthened. Consequently, a robust catalyst for overall water splitting was obtained, and it may also establish new directions for designing robust materials for energy conversion and storage in wide conditions.

## Supplementary Information

Below is the link to the electronic supplementary material.

Supplementary file2 (PDF 1578 KB)

## References

[1] J. Yang, A.R. Mohmad, Y. Wang, R. Fullon, X. Song et al., Ultrahigh-current-density niobium disulfide catalysts for hydrogen evolution. Nat. Mater. **18**, 1309–1314 (2019). 10.1038/s41563-019-0463-8 31451781 10.1038/s41563-019-0463-8

[2] S. Liu, J. Liu, X. Liu, J. Shang, L. Xu et al., Hydrogen storage in incompletely etched multilayer Ti_2_CT_*x*_ at room temperature. Nat. Nanotechnol. **16**, 331–336 (2021). 10.1038/s41565-020-00818-8 33398176 10.1038/s41565-020-00818-8

[3] D. Yan, C. Mebrahtu, S. Wang, R. Palkovits, Innovative electrochemical strategies for hydrogen production: from electricity input to electricity output. Angew. Chem. Int. Ed. **62**, e202214333 (2023). 10.1002/anie.202214333 10.1002/anie.202214333 36437229

[4] Q. Zhou, C. Xu, J. Hou, W. Ma, T. Jian et al., Duplex interpenetrating-phase FeNiZn and FeNi_3_ heterostructure with low-gibbs free energy interface coupling for highly efficient overall water splitting. Nano-Micro Lett. **15**, 95 (2023). 10.1007/s40820-023-01066-w 10.1007/s40820-023-01066-w PMC10086094 37037951

[5] H. Song, M. Wu, Z. Tang, J.S. Tse, B. Yang et al., Single atom ruthenium-doped CoP/CDs nanosheets via splicing of carbon-dots for robust hydrogen production. Angew. Chem. Int. Ed. **60**, 7234–7244 (2021). 10.1002/anie.202017102 10.1002/anie.202017102 33438321

[6] K. Yu, H. Yang, H. Zhang, H. Huang, Z. Wang et al., Immobilization of oxyanions on the reconstructed heterostructure evolved from a bimetallic oxysulfide for the promotion of oxygen evolution reaction. Nano-Micro Lett. **15**, 186 (2023). 10.1007/s40820-023-01164-9 10.1007/s40820-023-01164-9 PMC10387036 37515724

[7] D. Yan, C. Xia, W. Zhang, Q. Hu, C. He et al., Cation defect engineering of transition metal electrocatalysts for oxygen evolution reaction. Adv. Energy Mater. **12**, 2202317 (2022). 10.1002/aenm.202202317

[8] C. Li, S.H. Kim, H.Y. Lim, Q. Sun, Y. Jiang et al., Self-accommodation induced electronic metal-support interaction on ruthenium site for alkaline hydrogen evolution reaction. Adv. Mater. **35**, e2301369 (2023). 10.1002/adma.202301369 36853204 10.1002/adma.202301369

[9] J. Xu, Y. Feng, P. Wu, S. Tian, Z. Fang et al., Embedded ruthenium nanoparticles within exfoliated nanosheets of Ti_3_C_2_T_*x*_ for hydrogen evolution. ACS Appl. Nano Mater. **5**, 14241–14245 (2022). 10.1021/acsanm.2c03648

[10] B. Guo, Y. Ding, H. Huo, X. Wen, X. Ren et al., Recent advances of transition metal basic salts for electrocatalytic oxygen evolution reaction and overall water electrolysis. Nano-Micro Lett. **15**, 57 (2023). 10.1007/s40820-023-01038-0 10.1007/s40820-023-01038-0 PMC9981861 36862225

[11] X. Kong, C. Zhang, S.Y. Hwang, Q. Chen, Z. Peng, Free-standing holey Ni(OH)_2_ nanosheets with enhanced activity for water oxidation. Small **13**, 1700334 (2017). 10.1002/smll.201700334 10.1002/smll.201700334 28544425

[12] H.N. Nong, T. Reier, H.-S. Oh, M. Gliech, P. Paciok et al., A unique oxygen ligand environment facilitates water oxidation in hole-doped IrNiO_*x*_ core–shell electrocatalysts. Nat. Catal. **1**, 841–851 (2018). 10.1038/s41929-018-0153-y

[13] T. Reier, H.N. Nong, D. Teschner, R. Schlögl, P. Strasser, Electrocatalytic oxygen evolution reaction in acidic environments–reaction mechanisms and catalysts. Adv. Energy Mater. **7**, 1601275 (2017). 10.1002/aenm.201601275

[14] Y. Li, T. Xu, Q. Huang, L. Zhu, Y. Yan et al., C_60_ fullerenol to stabilize and activate Ru nanoparticles for highly efficient hydrogen evolution reaction in alkaline media. ACS Catal. **13**, 7597–7605 (2023). 10.1021/acscatal.3c01610

[15] H. Shi, H. Liang, F. Ming, Z. Wang, Efficient overall water-splitting electrocatalysis using lepidocrocite VOOH hollow nanospheres. Angew. Chem. Int. Ed. **56**, 573–577 (2017). 10.1002/anie.201610211 10.1002/anie.201610211 27897374

[16] Q. Liang, Q. Li, L. Xie, H. Zeng, S. Zhou et al., Superassembly of surface-enriched Ru nanoclusters from trapping-bonding strategy for efficient hydrogen evolution. ACS Nano **16**, 7993–8004 (2022). 10.1021/acsnano.2c00901 35394286 10.1021/acsnano.2c00901

[17] L. Zhang, N. Jin, Y. Yang, X.-Y. Miao, H. Wang et al., Advances on axial coordination design of single-atom catalysts for energy electrocatalysis: a review. Nano-Micro Lett. **15**, 228 (2023). 10.1007/s40820-023-01196-1 10.1007/s40820-023-01196-1 PMC10575848 37831204

[18] C.-F. Li, T.-Y. Shuai, L.-R. Zheng, H.-B. Tang, J.-W. Zhao et al., The key role of carboxylate ligands in Ru@Ni-MOFs/NF in promoting water dissociation kinetics for effective hydrogen evolution in alkaline media. Chem. Eng. J. **451**, 138618 (2023). 10.1016/j.cej.2022.138618

[19] P. Bhanja, Y. Kim, B. Paul, Y.V. Kaneti, A.A. Alothman et al., Microporous nickel phosphonate derived heteroatom doped nickel oxide and nickel phosphide: efficient electrocatalysts for oxygen evolution reaction. Chem. Eng. J. **405**, 126803 (2021). 10.1016/j.cej.2020.126803

[20] N.L.W. Septiani, Y.V. Kaneti, Y. Guo, B. Yuliarto, X. Jiang et al., Holey assembly of two-dimensional iron-doped nickel-cobalt layered double hydroxide nanosheets for energy conversion application. ChemSusChem **13**, 1645–1655 (2020). 10.1002/cssc.201901364 31270940 10.1002/cssc.201901364

[21] Y. Guo, C. Zhang, J. Zhang, K. Dastafkan, K. Wang et al., Metal–organic framework-derived bimetallic NiFe selenide electrocatalysts with multiple phases for efficient oxygen evolution reaction. ACS Sustain. Chem. Eng. **9**, 2047–2056 (2021). 10.1021/acssuschemeng.0c06969

[22] X. Gu, M. Yu, S. Chen, X. Mu, Z. Xu et al., Coordination environment of Ru clusters with *in situ* generated metastable symmetry-breaking centers for seawater electrolysis. Nano Energy **102**, 107656 (2022). 10.1016/j.nanoen.2022.107656

[23] N. Han, W. Zhang, W. Guo, H. Pan, B. Jiang et al., Designing oxide catalysts for oxygen electrocatalysis: insights from mechanism to application. Nano-Micro Lett. **15**, 185 (2023). 10.1007/s40820-023-01152-z 10.1007/s40820-023-01152-z PMC10387042 37515746

[24] D. Wu, D. Chen, J. Zhu, S. Mu, Ultralow Ru incorporated amorphous cobalt-based oxides for high-current-density overall water splitting in alkaline and seawater media. Small **17**, e2102777 (2021). 10.1002/smll.202102777 34390190 10.1002/smll.202102777

[25] G. Li, H. Jang, S. Liu, Z. Li, M.G. Kim et al., The synergistic effect of Hf-O-Ru bonds and oxygen vacancies in Ru/HfO_2_ for enhanced hydrogen evolution. Nat. Commun. **13**, 1270 (2022). 10.1038/s41467-022-28947-9 35277494 10.1038/s41467-022-28947-9 PMC8917135

[26] J. Xu, S. Wang, C. Yang, T. Li, Q. Liu et al., Free-standing two-dimensional ruthenium-beryllium nanosheets for alkaline hydrogen evolution. Chem. Eng. J. **421**, 129741 (2021). 10.1016/j.cej.2021.129741

[27] S. Li, D. Liu, G. Wang, P. Ma, X. Wang et al., Vertical 3D nanostructures boost efficient hydrogen production coupled with glycerol oxidation under alkaline conditions. Nano-Micro Lett. **15**, 189 (2023). 10.1007/s40820-023-01150-1 10.1007/s40820-023-01150-1 PMC10387032 37515627

[28] F. Bao, Z. Yang, Y. Yuan, P. Yu, G. Zeng et al., Synergistic cascade hydrogen evolution boosting via integrating surface oxophilicity modification with carbon layer confinement. Adv. Funct. Mater. **32**, 2108991 (2022). 10.1002/adfm.202108991

[29] S. Hao, M. Liu, J. Pan, X. Liu, X. Tan et al., Dopants fixation of Ruthenium for boosting acidic oxygen evolution stability and activity. Nat. Commun. **11**, 5368 (2020). 10.1038/s41467-020-19212-y 33097730 10.1038/s41467-020-19212-y PMC7584605

[30] A. Grimaud, O. Diaz-Morales, B. Han, W.T. Hong, Y.-L. Lee et al., Activating lattice oxygen redox reactions in metal oxides to catalyse oxygen evolution. Nat. Chem. **9**, 457–465 (2017). 10.1038/nchem.2695 28430191 10.1038/nchem.2695

[31] P. Gayen, S. Saha, K. Bhattacharyya, V.K. Ramani, Oxidation state and oxygen-vacancy-induced work function controls bifunctional oxygen electrocatalytic activity. ACS Catal. **10**, 7734–7746 (2020). 10.1021/acscatal.0c01541

[32] C. Roy, R.R. Rao, K.A. Stoerzinger, J. Hwang, J. Rossmeisl et al., Trends in activity and dissolution on RuO_2_ under oxygen evolution conditions: particles versus well-defined extended surfaces. ACS Energy Lett. **3**, 2045–2051 (2018). 10.1021/acsenergylett.8b01178

[33] S. Chen, H. Huang, P. Jiang, K. Yang, J. Diao et al., Mn-doped RuO_2_ nanocrystals as highly active electrocatalysts for enhanced oxygen evolution in acidic media. ACS Catal. **10**, 1152–1160 (2020). 10.1021/acscatal.9b04922

[34] J. Su, R. Ge, K. Jiang, Y. Dong, F. Hao et al., Assembling ultrasmall copper-doped ruthenium oxide nanocrystals into hollow porous polyhedra: highly robust electrocatalysts for oxygen evolution in acidic media. Adv. Mater., e1801351 (2018). 10.1002/adma.201801351 10.1002/adma.201801351 29870585

[35] X. Cui, P. Ren, C. Ma, J. Zhao, R. Chen et al., Robust interface Ru centers for high-performance acidic oxygen evolution. Adv. Mater. **32**, e1908126 (2020). 10.1002/adma.201908126 32419157 10.1002/adma.201908126

[36] J. Wang, H. Yang, F. Li, L. Li, J. Wu et al., Single-site Pt-doped RuO_2_ hollow nanospheres with interstitial C for high-performance acidic overall water splitting. Sci. Adv. **8**, eabl9271 (2022). 10.1126/sciadv.abl9271 10.1126/sciadv.abl9271 PMC8890715 35235348

[37] M.A. Hubert, A.M. Patel, A. Gallo, Y. Liu, E. Valle et al., Acidic oxygen evolution reaction activity–stability relationships in Ru-based pyrochlores. ACS Catal. **10**, 12182–12196 (2020). 10.1021/acscatal.0c02252

[38] D. Zhang, M. Li, X. Yong, H. Song, G.I.N. Waterhouse et al., Construction of Zn-doped RuO_2_ nanowires for efficient and stable water oxidation in acidic media. Nat. Commun. **14**, 2517 (2023). 10.1038/s41467-023-38213-1 37130878 10.1038/s41467-023-38213-1 PMC10154325

[39] A.M. Harzandi, S. Shadman, A.S. Nissimagoudar, D.Y. Kim, H.-D. Lim et al., Ruthenium core–shell engineering with nickel single atoms for selective oxygen evolution via nondestructive mechanism. Adv. Energy Mater. **11**, 2003448 (2021). 10.1002/aenm.202003448

[40] Y. Zhang, G.-Q. Mao, X. Zhao, Y. Li, M. Zhang et al., Evolution of the conductive filament system in HfO_2_-based memristors observed by direct atomic-scale imaging. Nat. Commun. **12**, 7232 (2021). 10.1038/s41467-021-27575-z 34903752 10.1038/s41467-021-27575-z PMC8668918

[41] D. Huang, K. Wang, L. Yu, T.H. Nguyen, S. Ikeda et al., Over 1% efficient unbiased stable solar water splitting based on a sprayed Cu_2_ZnSnS_4_ photocathode protected by a HfO_2_ photocorrosion-resistant film. ACS Energy Lett. **3**, 1875–1881 (2018). 10.1021/acsenergylett.8b01005

[42] W. Banerjee, A. Kashir, S. Kamba, Hafnium oxide (HfO_2_) - A multifunctional oxide: a review on the prospect and challenges of hafnium oxide in resistive switching and ferroelectric memories. Small **18**, e2107575 (2022). 10.1002/smll.202107575 35510954 10.1002/smll.202107575

[43] Y. Wang, Q. Lu, F. Li, D. Guan, Y. Bu, Atomic-scale configuration enables fast hydrogen migration for electrocatalysis of acidic hydrogen evolution. Adv. Funct. Mater. **33**, 2213523 (2023). 10.1002/adfm.202213523

[44] J. Xu, C. Chen, X. Kong, Ru-O-Cu center constructed by catalytic growth of Ru for efficient hydrogen evolution. Nano Energy **111**, 108403 (2023). 10.1016/j.nanoen.2023.108403

[45] J. Xu, X. Kong, Amorphous/crystalline heterophase ruthenium nanosheets for pH-universal hydrogen evolution. Small Methods **6**, e2101432 (2022). 10.1002/smtd.202101432 34957700 10.1002/smtd.202101432

[46] P. Wu, X. Kong, Y. Feng, W. Ding, Z. Sheng et al., Phase engineering on amorphous/crystalline γ-Fe_2_O_3_ nanosheets for boosting dielectric loss and high-performance microwave absorption. Adv. Funct. Mater., **34**, 2311983 (2024). 10.1002/adfm.202311983

[47] G. Wu, X. Zheng, P. Cui, H. Jiang, X. Wang et al., A general synthesis approach for amorphous noble metal nanosheets. Nat. Commun. **10**, 4855 (2019). 10.1038/s41467-019-12859-2 31649272 10.1038/s41467-019-12859-2 PMC6813339

[48] G. Yan, Y. Wang, Z. Zhang, Y. Dong, J. Wang et al., Nanoparticle-decorated ultrathin La_2_O_3_ nanosheets as an efficient electrocatalysis for oxygen evolution reactions. Nano-Micro Lett. **12**, 49 (2020). 10.1007/s40820-020-0387-5 10.1007/s40820-020-0387-5 PMC7770806 34138270

[49] M. Su, J. Shi, Q. Kang, D. Lai, Q. Lu et al., One-step multiple structure modulations on sodium vanadyl phosphate@carbon towards ultra-stable high rate sodium storage. Chem. Eng. J. **432**, 134289 (2022). 10.1016/j.cej.2021.134289

[50] A. Pei, G. Li, L. Zhu, Z. Huang, J. Ye et al., Nickel hydroxide-supported Ru single atoms and Pd nanoclusters for enhanced electrocatalytic hydrogen evolution and ethanol oxidation. Adv. Funct. Mater. **32**, 2208587 (2022). 10.1002/adfm.202208587

[51] L. Cao, Q. Luo, J. Chen, L. Wang, Y. Lin et al., Dynamic oxygen adsorption on single-atomic Ruthenium catalyst with high performance for acidic oxygen evolution reaction. Nat. Commun. **10**, 4849 (2019). 10.1038/s41467-019-12886-z 31649237 10.1038/s41467-019-12886-z PMC6813412

[52] D. Majumdar, T. Maiyalagan, Z. Jiang, Recent progress in ruthenium oxide-based composites for supercapacitor applications. ChemElectroChem **6**, 4343–4372 (2019). 10.1002/celc.201900668

[53] Y. Hu, C. Hu, A. Du, T. Xiao, L. Yu et al., Interfacial evolution on co-based oxygen evolution reaction electrocatalysts probed by using *in situ* surface-enhanced Raman spectroscopy. Anal. Chem. **95**, 1703–1709 (2023). 10.1021/acs.analchem.2c04931 36583685 10.1021/acs.analchem.2c04931

[54] J.C. Dong, M. Su, V. Briega-Martos, L. Li, J.B. Le et al., Direct *in situ* Raman spectroscopic evidence of oxygen reduction reaction intermediates at high-index Pt(*hkl*) surfaces. J. Am. Chem. Soc. **142**, 715–719 (2020). 10.1021/jacs.9b12803 31887023 10.1021/jacs.9b12803

