# Supplementary material for: Durable Ru Nanocrystal with HfO2 Modification for Acidic Overall Water Splitting
Source: Nanomicro Lett. 2024 Apr 30;16:185. doi: 10.1007/s40820-024-01384-7 (PMC11061093; doi:10.1007/s40820-024-01384-7)
Supplement: Supplementary file 1 — Supplementary file2 (PDF 1578 KB) [file 40820_2024_1384_MOESM1_ESM.pdf]

Supporting Information for

Durable Ru Nanocrystal with HfO_2 Modification for Acidic Overall Water Splitting

Xiangkai Kong[1, 2, *], Jie Xu[2, 4], Zhicheng Ju[1, *], and Changle Chen[3, *]

[1] School of Materials and Physics, China University of Mining and Technology, Xuzhou, Jiangsu 221116, P. R. China

[2] Anhui Province Key Laboratory of Pollutant Sensitive Materials and Environmental Remediation, Huaibei Normal University, Huaibei, Anhui 235000, P. R. China

[3] School of Chemistry and Materials Science, University of Science and Technology of China, Hefei, Anhui 230026, P. R. China

[4] School of Chemistry and Chemical Engineering, Nanjing University, Nanjing, Jiangsu 210023, P. R. China

*Corresponding authors. E-mail: changle@ustc.edu.cn (Changle Chen), xkong@cumt.edu.cn (Xiangkai Kong), juzc@cumt.edu.cn (Zhicheng Ju)

Supplementary Figures and Tables

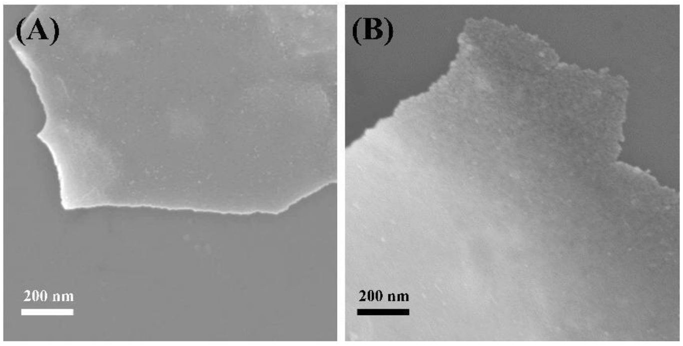

Fig. S1 SEM images of S-Ru/HfO_2

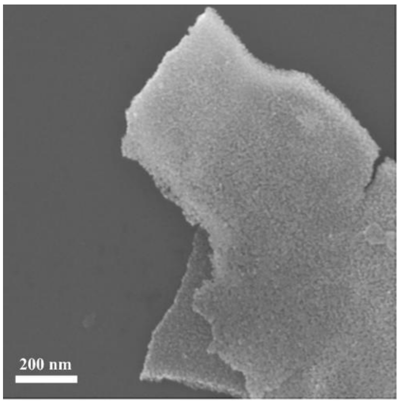

Fig. S2 SEM image of L-Ru/HfO_2

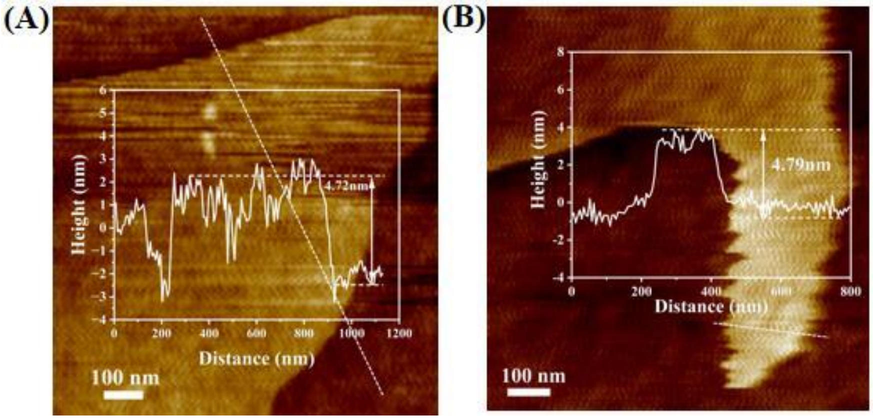

Fig. S3 The height profiles of individual nanosheets for (**A**) L-Ru/HfO_2 and (**B**) S-Ru/HfO_2

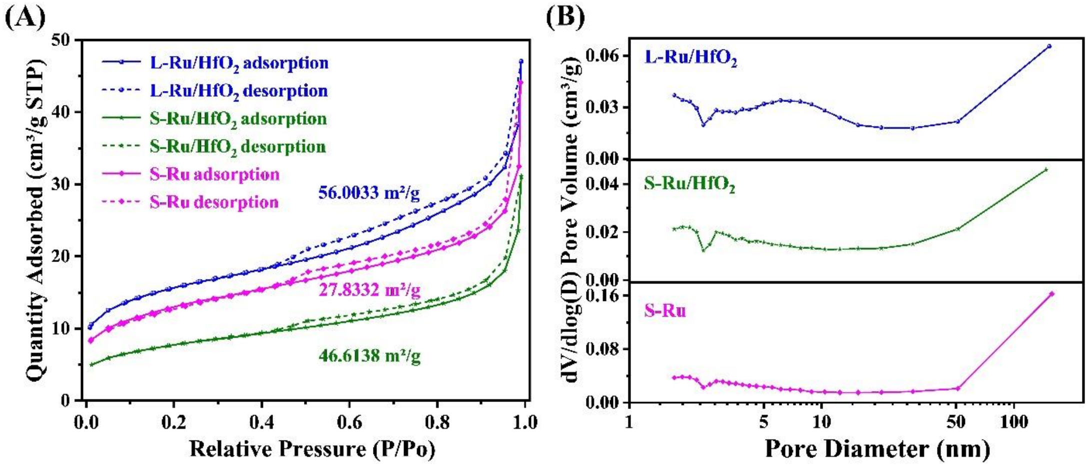

Fig. S4 BET data of (**A**) surface area and (**B**) pore size distribution for these samples

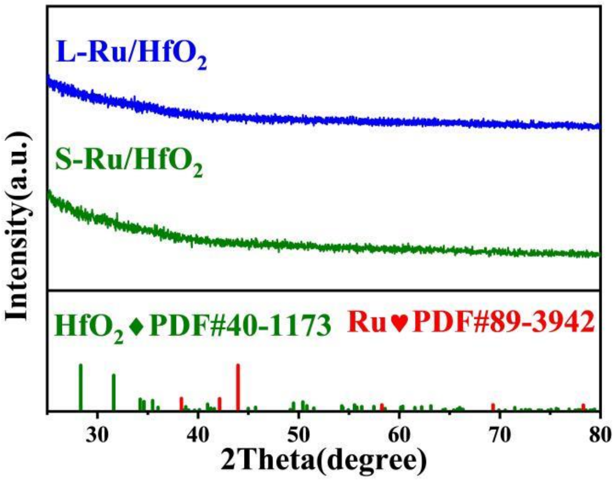

Fig. S5 XRD patterns of L-Ru/HfO_2 and S-Ru/HfO_2

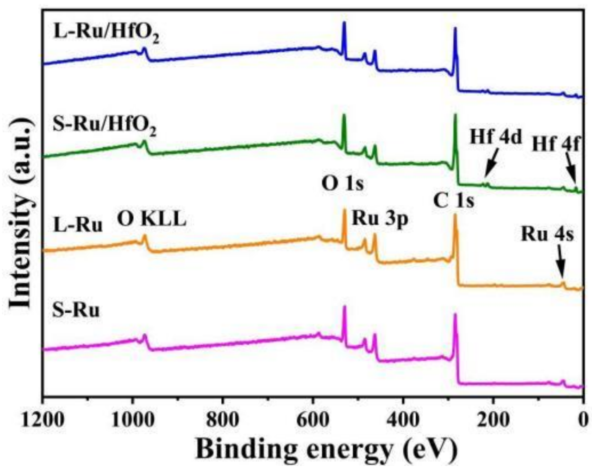

Fig. S6 XPS survey spectra of these as-synthesized samples

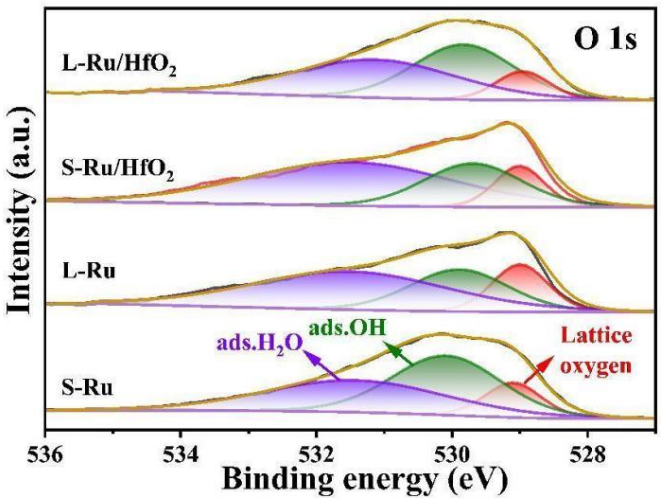

Fig. S7 O 1s spectra of these as-synthesized samples

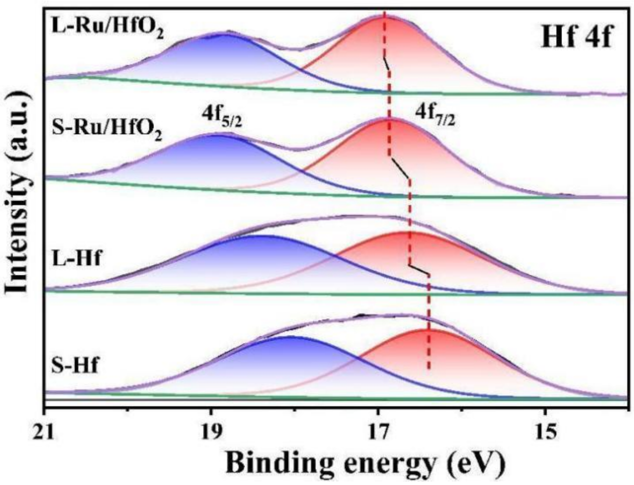

Fig. S8 Hf 4f spectra of these as-synthesized samples

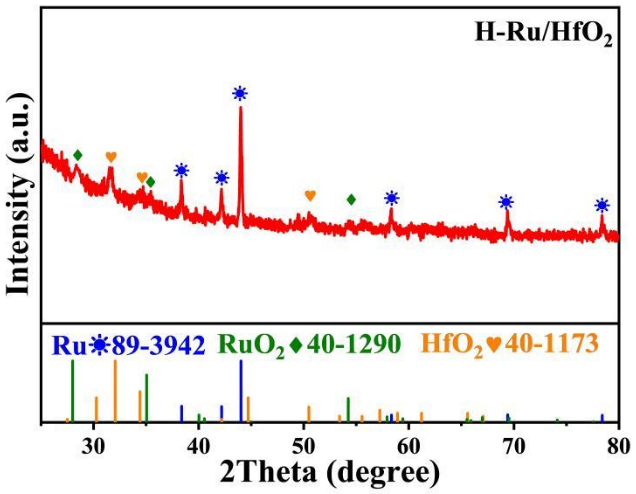

Fig. S9 XRD pattern of H-Ru/HfO_2

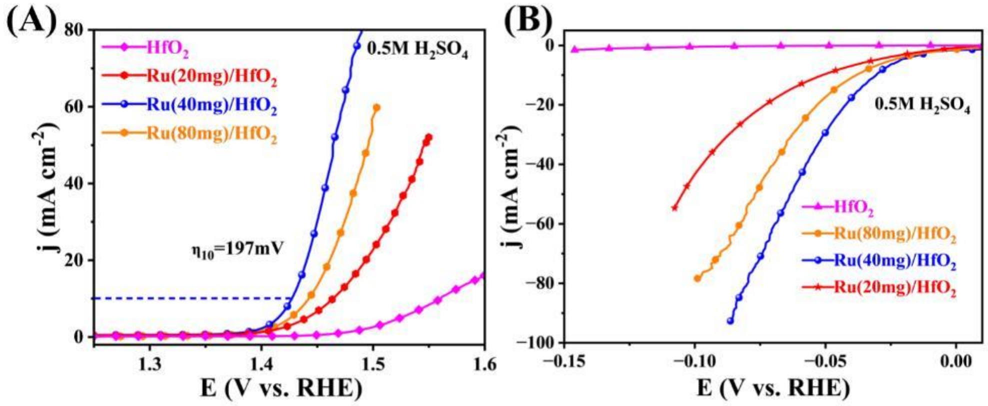

Fig. S10 The LSV curves of acidic (**A**) OER and (**B**) HER recorded on samples prepared with different amounts of Ru(acac)$_3$

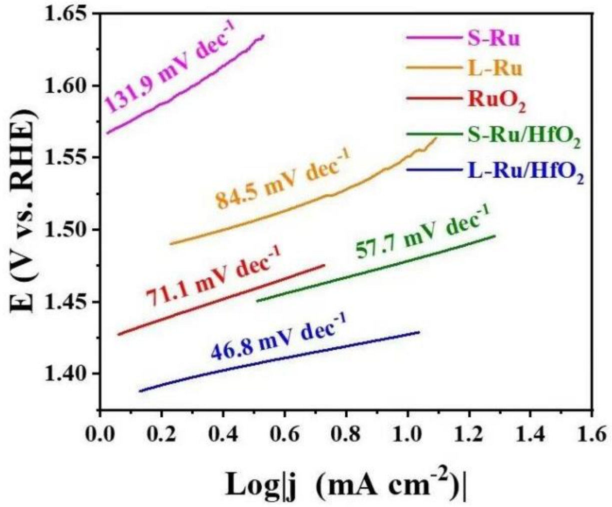

Fig. S11 Tafel slopes of these catalysts for OER measurement in 0.5 M H_2SO_4

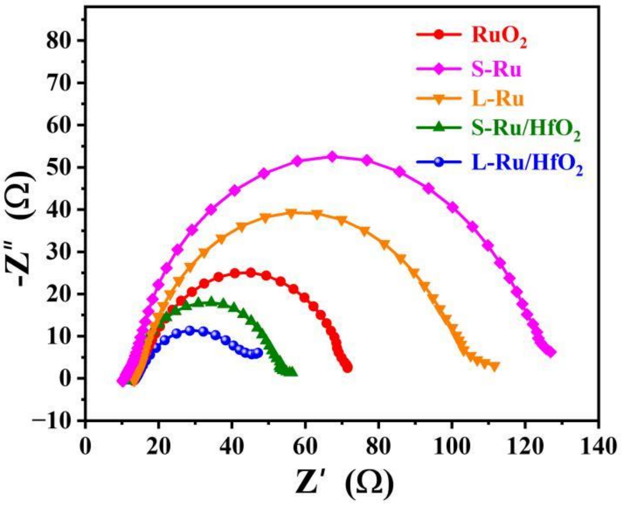

Fig. S12 EIS of these catalysts recorded at an overpotential of 200mV for OER in 0.5 M H_2SO_4

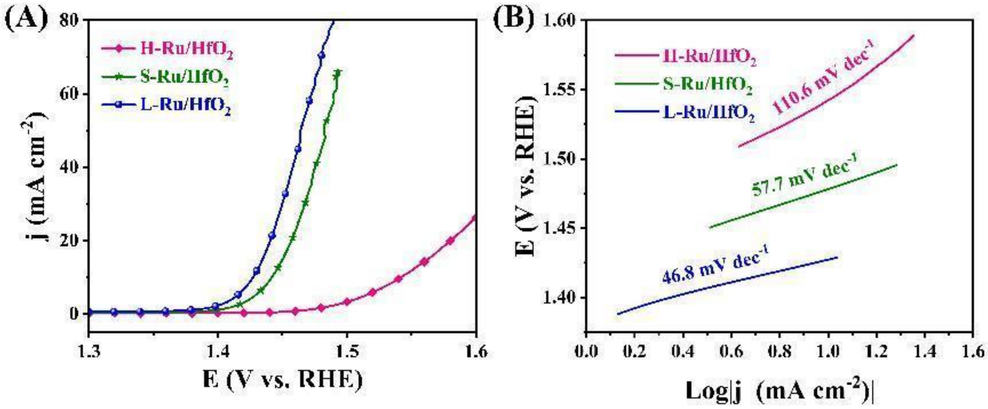

Fig. S13 Electrochemical measurement in 0.5 M H_2SO_4 for comparison of H-Ru/HfO_2 with L-Ru/HfO_2 and S-Ru/HfO_2: (**A**) LSV curves and (**B**) Tafel slopes.

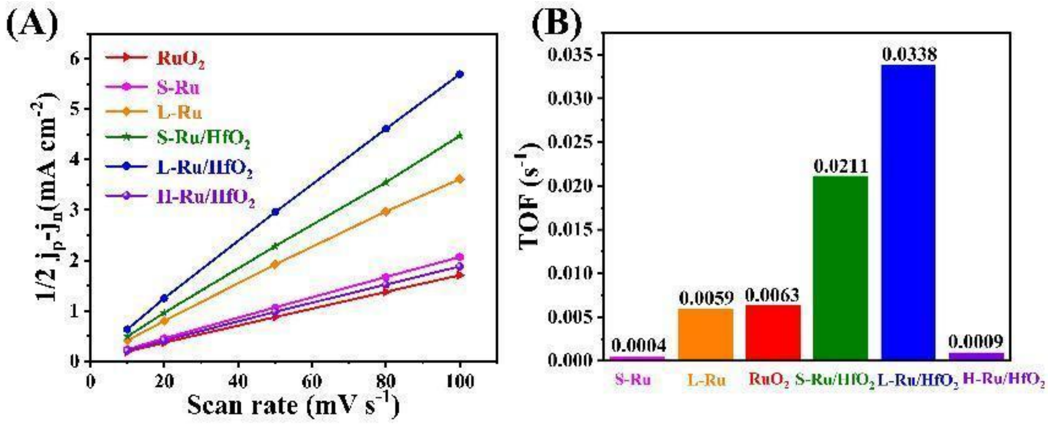

Fig. S14 (**A**) The current difference between positive scan and negative scan as a function of scan rates for double layer capacitance evaluation. (**B**) The calculated TOFs for these samples

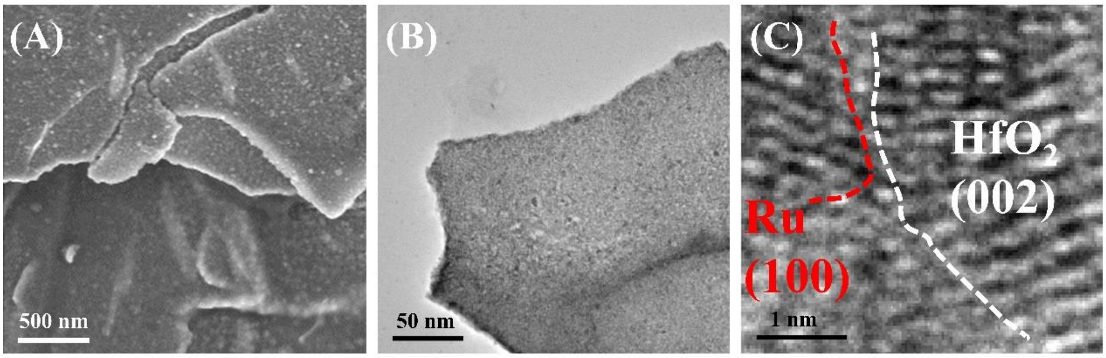

Fig. S15 The physical characterizations on L-Ru/HfO_2 after the durability test: (**A**) SEM, (**B**) TEM and (**C**) HRTEM images

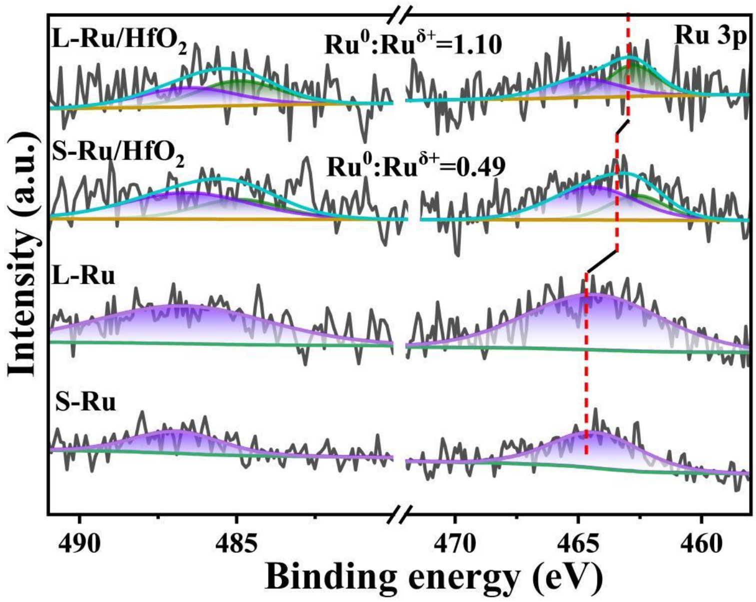

Fig. S16 XPS spectra of Ru 3p signals of these samples after the durability test

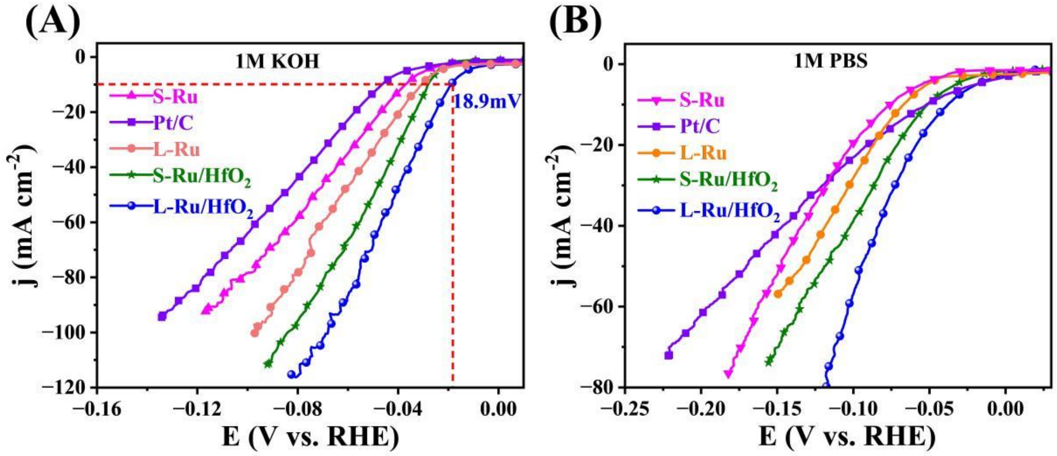

Fig. S17 The measured LSV curves of these samples during HER operation in (**A**) 1 M KOH and (**B**) 1 M PBS electrolytes

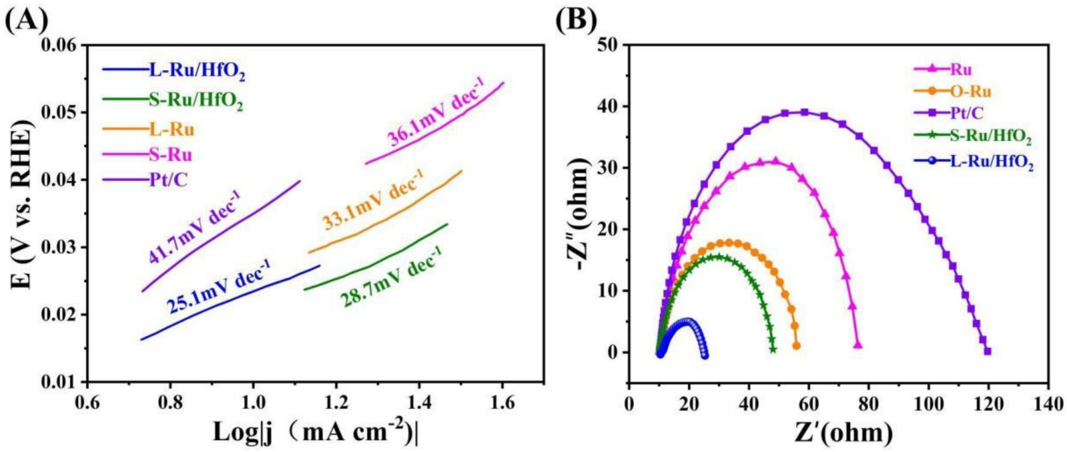

Fig. S18 The obtained (A) Tafel slopes and (B) EIS curves of these samples for HER measurement

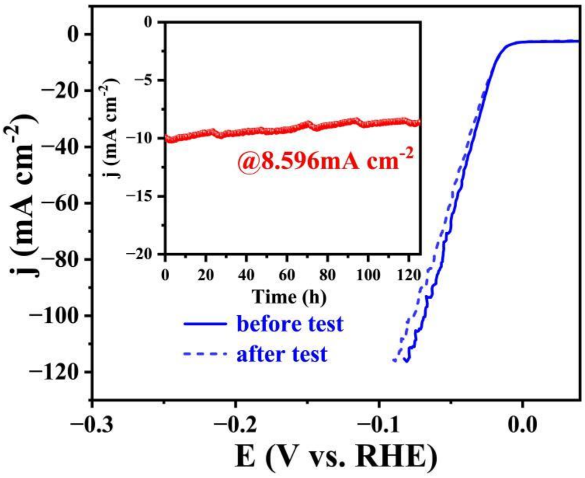

Fig. S19 The chronoamperometry measurement of L-Ru/HfO_2 for HER stability evaluation, with the LSV curves before and after the stability test inset in

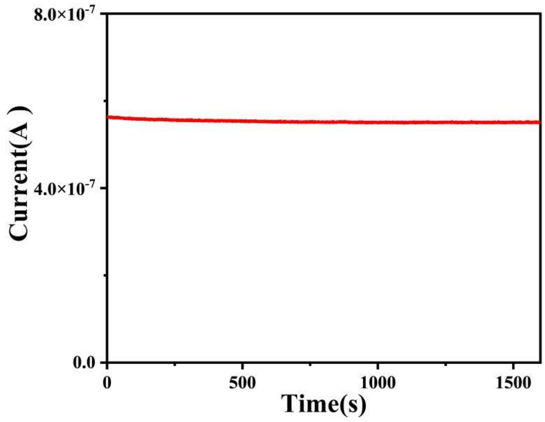

Fig. S20 The recorded current during the in situ XPS measurement with an applied voltage of 2.0 V

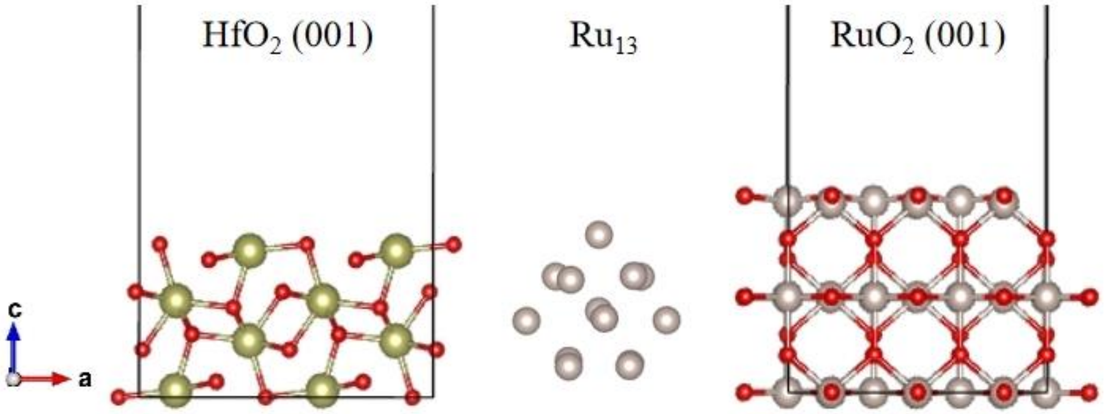

Fig. S21 The simulated models for (**A**) HfO_2 (001) surface, (**B**) Ru_{13} cluster and (**C**) RuO_2 (001) surface

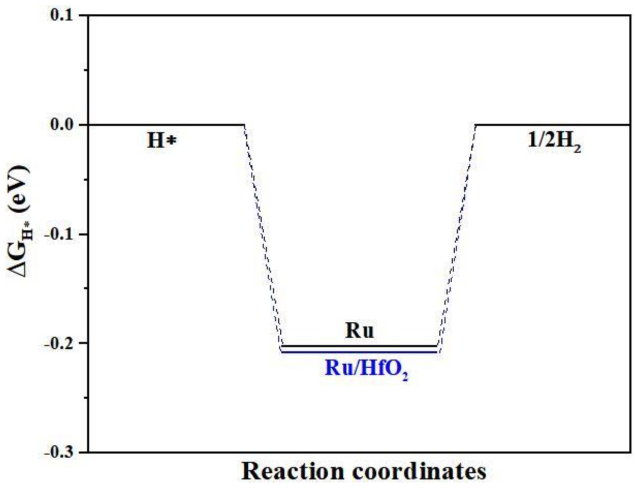

Fig. S22 HER free-energy diagrams of Ru and Ru/HfO_2 for comparison

Table S1 Ru *K*-edge EXAFS least-squares fitting parameters[a] for Ru foil, RuO_2 standard, 464-Ru-1, and 464-Ru-2 sample

Material	Path	N	*R* (Å)	σ^2 (Å^2)	ΔE_0 (eV)	*R*-factor
Ru foil[b]	Ru-Ru	12	2.351±0.038	0.00290	-6.95±0.64	0.159%
RuO_2[c]	Ru-O1	6	1.575±0.095	0.00297	-5.75±0.78	0.167%
	Ru-O2	12	1.482±0.020	0.00281		
S-Ru/HfO_2[d]	Ru-O-Hf	0.82±0.05	1.729±0.066	0.00180	5.25±0.38	0.657%
	Ru-Ru	4.65±0.29	2.416±0.050	0.00597		
L-Ru/HfO_2[d]	Ru-O-Hf	0.76±0.08	1.776±0.003	0.00127	5.79±0.50	0.880%
	Ru-Ru	6.61±0.48	2.388±0.042	0.00633		

[a]N, coordination number; R, distance between absorber and backscatter atoms; σ^2, Debye-Waller factor to account for both thermal and structural disorders; ΔE_0, inner potential correction; R-factor (%) generally estimates the goodness of the fit. Error bounds (accuracies) that characterize the structural parameters obtained by EXAFS spectroscopy were estimated as N±20%; R±1%; σ^2±20%; ΔE_0±20%. S_0^2 was fixed as 0.80, which was determined by fitting the experimental data on Ru foil with fixed coordination numbers (in bold) according to the crystal structure, as well for RuO_2 standard. [b]Fitting range for Ru foil was selected to be $2.0 \leq k \leq 12.2$ Å^{-1} (k^2-weighted) and $1.7 \leq R \leq 3.0$ Å, yielding the number of variable parameters being 3, out of a total of 8.13 independent data points. [c]Fitting range for RuO_2 was selected to be $2.0 \leq k \leq 13.5$ Å^{-1} (k^2-weighted) and $1.0 \leq R \leq 2.0$ Å, yielding the number of variable parameters being 3, out of a total of 7.16 independent data points. [d]Fitting range for Ru sample was selected to be $2.8 \leq k \leq 9.5$ Å^{-1} (k^3-weighted) and $1.0 \leq R \leq 3.0$ Å, yielding the number of variable parameters being 4, out of a total of 8.31 independent data points.

Table S2 Comparison of OER activity of L-Ru/HfO_2 with different catalysts

Catalyst	η_{10} (mV)	Tafel plots (mV dec^{-1})	Electrolyte	Refs.
L-Ru/HfO_2	197	46.8	0.5M H_2SO_4	this work
Co-RuO_2/OCNT	260	83	0.1 M KOH	S1
E-Ru/Fe ONAs	238	44.8	0.5M H_2SO_4	S2
Ru-UiO-67-bpydc	200	78.3	0.5M H_2SO_4	S3
RuO_2/$(Co,Mn)_3O_4$/CC	270	77	0.5M H_2SO_4	S4
L-Ru	202	69.6	0.5M H_2SO_4	S5
Ru SAs/AC-FeCoNi	205	40	1 M KOH	S6
3%Rh-FeOOH@$Ti_3C_2T_x$	223	63.6	1 M KOH	S7
Ru NCs/VN-C_3N_4	200	60	1 M KOH	S8
Ru1Ir1O_x	240	71.3	0.5M H_2SO_4	S9
$Mn_{0.73}Ru_{0.27}O_{2-\delta}$	208	65.3	0.5M H_2SO_4	S10
$RuNi_2$©G-250	227	65	0.5M H_2SO_4	S11
Ru/Co_3O_{4-x}	280	86.9	1 M KOH	S12
Ru/S NSs-400	219	46.1	0.5M H_2SO_4	S13
IrRu@WO_3	245	62	0.5M H_2SO_4	S14
Ru@g-CN_x	280	49.5	1 M KOH	S15
HS-RuCo/NC	216	76.1	1 M KOH	S16
Ni_3Co_3@Ru HNS	300	60	0.1 M KOH	S17
Ru/NiFe(OH)x/NiFe-MOF	242	30.63	1 M KOH	S18
$Ru_{0.6}W_{17}.4O_{49-\delta}$	252	50	0.1 M HClO4	S19
Ru/Co–N–C	232	67.5	0.5M H_2SO_4	S20
S-F-400	241	56	1 M KOH	S21

Ru1/VCo-Co(OH)$_2$	241	74	1 M KOH	S22
Ni-Ru@RuO$_x$-HL	184	44	0.5M H_2SO_4	S23
CoO$_x$/RuOx-CC	180	61.2	0.5M H_2SO_4	S24
Ru-exchanged Cu-BTC	188	43.96	0.5M H_2SO_4	S25
IrRu@Te	220	35	0.5M H_2SO_4	S26
LFRO-H-O	380	39	1 M KOH	S27
Ru-N-C	267	52.6	0.5M H_2SO_4	S28
YZRO/AB	291	36.9	0.5M H_2SO_4	S29
Ru–NiSe$_2$/NF	210	60.5	1 M KOH	S30
STRO/NF	375	224	0.1 M KOH	S31
PRPO-350	174	28.8	0.1 M HClO4	S32
NaAl-LRNO	270	69.3	1 M KOH	S33
Ru/Se-RuO$_2$	190	43.7	0.5M H_2SO_4	S34
Ru$_x$ SACs@FeCo-LDH	194	25	1 M KOH	S35
Co-Fe-Ru/PNCS	310	84.6	1 M KOH	S36
$Bi_2Ru_2O_7$	448	108	0.1 M KOH	S37
Sn$_{0.1}$-RuO$_2$@NCP	178	60.6	0.5M H_2SO_4	S38
Mo–YRO	240	40.5	0.1 M HClO4	S39
Ru/NiFe^{2+}Fe-LDH	194	36	1 M KOH	S40
NiFeRu-LDH	225	32.4	1 M KOH	S41
CIS@Ir$_{48}$Ru$_{52}$	244.4	68.4	0.1 M HClO4	S42
RuO$_2$ NS$_s$	199	38.2	0.5M H_2SO_4	S43
RuCo@CD	190	49.5	0.5M H_2SO_4	S44
RuTe$_2$-400	275	53	1 M KOH	S45
Ru$_3$MoCeO$_x$	164	61.2	0.5M H_2SO_4	S46
CoNiRuO$_x$-2	245	82.3	1 M KOH	S47
NiRuOx-C	215	52.6	1 M KOH	S48
Co-Ru@RuOx/NCN	270	67	1 M KOH	S49
6.8% Rh SAC-CuO NA$_s$/CF	197	71.7	1 M KOH	S50
3D Se-(NiCo)S$_x$/(OH)$_x$	155	33.9	1 M KOH	S51
NiIrRuAl-1/3	237	50	0.1 M HClO4	S52
RuIrTe NTs	205	41.2	0.5M H_2SO_4	S53
In$_{0.17}$Ru$_{0.83}$O$_2$-35	177	32.62	0.5 M H2SO4	S54
Ru/B-Ni$_2$P/Ni$_5$P$_4$	270	46.7	1 M KOH	S55
AlNiCoRuMoCrFeTiHEO	240	51.3	1 M KOH	S56
Ru$_{9.1}$-NiFe-MOF/NFF	202	30.5	1 M KOH	S57
RuO$_2$/CoO$_x$	240	70	1 M PBS	S58

Supplementary References

[S1] Q. Lu, X. Zou, X. Wang, L. An, Z. Shao et al., Simultaneous reactant accessibility and charge transfer engineering in Co-doped RuO2-supported OCNT for robust rechargeable zinc-air batteries. Appl. Catal. B Environ. 325, 122323 (2023). https://doi.org/10.1016/j.apcatb.2022.122323

[S2] Q. Yao, B. Huang, Y. Xu, L. Li, Q. Shao et al., A chemical etching strategy to improve and stabilize RuO_2-based nanoassemblies for acidic oxygen evolution. Nano Energy **84**, 105909 (2021). https://doi.org/10.1016/j.nanoen.2021.105909

[S3] N. Yao, H. Jia, J. Zhu, Z. Shi, H. Cong et al., Atomically dispersed Ru oxide catalyst with lattice oxygen participation for efficient acidic water oxidation. Chem **9**, 1882–1896 (2023). https://doi.org/10.1016/j.chempr.2023.03.005

[S4] S. Niu, X.-P. Kong, S. Li, Y. Zhang, J. Wu et al., Low Ru loading RuO_2/(Co, Mn)$_3O_4$ nanocomposite with modulated electronic structure for efficient oxygen evolution reaction in acid. Appl. Catal. B Environ. **297**, 120442 (2021). https://doi.org/10.1016/j.apcatb.2021.120442

[S5] J.-Q. Wang, C. Xi, M. Wang, L. Shang, J. Mao et al., Laser-generated grain boundaries in ruthenium nanoparticles for boosting oxygen evolution reaction. ACS Catal. **10**, 12575–12581 (2020). https://doi.org/10.1021/acscatal.0c03406

[S6] Y. Hu, G. Luo, L. Wang, X. Liu, Y. Qu et al., Single Ru atoms stabilized by hybrid amorphous/crystalline FeCoNi layered double hydroxide for ultraefficient oxygen evolution. Adv. Energy Mater. **11**, 2002816 (2021). https://doi.org/10.1002/aenm.202002816

[S7] B. Zhang, J. Shan, X. Wang, Y. Hu, Y. Li Ru/Rh cation doping and oxygen-vacancy engineering of FeOOH Nanoarrays@Ti_3 C_2 t_x MXene heterojunction for highly efficient and stable electrocatalytic oxygen evolution. Small **18**, e2200173 (2022). https://doi.org/10.1002/smll.202200173

[S8] J. Zhao, H. Guo, Y. Li, L. Zheng, H. Ren et al., Anchoring Ru nanoclusters to defect-rich polymeric carbon nitride as a bifunctional electrocatalyst for highly efficient overall water splitting. J. Mater. Chem. A **11**, 18375–18386 (2023). https://doi.org/10.1039/d3ta02817h

[S9] J. He, X. Zhou, P. Xu, J. Sun Regulating electron redistribution of intermetallic iridium oxide by incorporating Ru for efficient acidic water oxidation. Adv. Energy Mater. **11**, 2102883 (2021). https://doi.org/10.1002/aenm.202102883

[S10] K. Wang, Y. Wang, B. Yang, Z. Li, X. Qin et al., Highly active ruthenium sites stabilized by modulating electron-feeding for sustainable acidic oxygen-evolution electrocatalysis. Energy Environ. Sci. **15**, 2356–2365 (2022). https://doi.org/10.1039/D1EE03610F

[S11] X. Cui, P. Ren, C. Ma, J. Zhao, R. Chen et al., Robust interface Ru centers for high-performance acidic oxygen evolution. Adv. Mater. **32**, e1908126 (2020). https://doi.org/10.1002/adma.201908126

[S12] C.-Z. Yuan, S. Wang, K. San Hui, K. Wang, J. Li et al., *In situ* immobilizing atomically dispersed Ru on oxygen-defective Co_3O_4 for efficient oxygen evolution. ACS Catal. **13**, 2462–2471 (2023). https://doi.org/10.1021/acscatal.2c04946

[S13] L. Liu, Y. Ji, W. You, S. Liu, Q. Shao et al., Trace lattice S inserted RuO_2 flexible nanosheets for efficient and long-term acidic oxygen evolution catalysis. Small **19**, e2208202 (2023). https://doi.org/10.1002/smll.202208202

[S14] G. Jiang, H. Yu, D. Yao, Y. Li, J. Chi et al., Boosting the oxygen evolution stability and activity of a heterogeneous IrRu bimetallic coating on a WO_3 nano-array electrode for PEM water electrolysis. J. Mater. Chem. A **10**, 11893–11903 (2022). https://doi.org/10.1039/d1ta09887j

[S15] T. Gao, K.S. Kumar, Z. Yan, M. Marinova, M. Trentesaux et al., Covalent organic framework derived synthesis of Ru embedded in carbon nitride for hydrogen and oxygen evolution reactions. J. Mater. Chem. A **11**, 19338–19348 (2023). https://doi.org/10.1039/D3TA01362F

[S16] J. Du, D. Chen, Y. Ding, L. Wang, F. Li et al., Highly stable and efficient oxygen evolution electrocatalyst based on co oxides decorated with ultrafine Ru nanoclusters. Small **19**, e2207611 (2023). https://doi.org/10.1002/smll.202207611

[S17] H. Hwang, T. Kwon, H.Y. Kim, J. Park, A. Oh et al., Ni@Ru and NiCo@Ru core-shell hexagonal nanosandwiches with a compositionally tunable core and a regioselectively grown shell. Small **14**. DOI: 10.1002/smll.201702353. (2018). https://doi.org/10.1002/smll.201702353

[S18] D. Liu, H. Xu, C. Wang, C. Ye, R. Yu et al., *In situ* etch engineering of Ru doped $NiFe(OH)_x$/NiFe-MOF nanocomposites for boosting the oxygen evolution reaction. J. Mater. Chem. A **9**, 24670–24676 (2021). https://doi.org/10.1039/d1ta06438j

[S19] X. Wang, H. Jang, S. Liu, Z. Li, X. Zhao et al., Enhancing the catalytic kinetics and stability of Ru sites for acidic water oxidation by forming Brønsted acid sites in tungsten oxide matrix. Adv. Energy Mater. **13**, 2301673 (2023). https://doi.org/10.1002/aenm.202301673

[S20] C. Rong, X. Shen, Y. Wang, L. Thomsen, T. Zhao et al., Electronic structure engineering of single-atom Ru sites via co-N4 sites for bifunctional pH-universal water splitting. Adv. Mater. **34**, e2110103 (2022). https://doi.org/10.1002/adma.202110103

[S21] K. Yang, P. Xu, Z. Lin, Y. Yang, P. Jiang et al., Ultrasmall Ru/Cu-doped RuO_2 complex embedded in amorphous carbon skeleton as highly active bifunctional

electrocatalysts for overall water splitting. Small **14**, e1803009 (2018). https://doi.org/10.1002/smll.201803009

[S22] D. Wang, J. Xue, X. Ding, J. Wei, C. Feng et al., Neighboring cationic vacancy assisted adsorption optimization on single-atom sites for improved oxygen evolution. ACS Catal. **12**, 12458–12468 (2022). https://doi.org/10.1021/acscatal.2c03476

[S23] A.M. Harzandi, S. Shadman, A.S. Nissimagoudar, D.Y. Kim, H.-D. Lim et al., Ruthenium core–shell engineering with nickel single atoms for selective oxygen evolution via nondestructive mechanism. Adv. Energy Mater. **11**, 2003448 (2021). https://doi.org/10.1002/aenm.202003448

[S24] L. Deng, S. Liu, D. Liu, Y.-M. Chang, L. Li et al., Activity-stability balance: the role of electron supply effect of support in acidic oxygen evolution. Small **19**, e2302238 (2023). https://doi.org/10.1002/smll.202302238

[S25] J. Su, R. Ge, K. Jiang, Y. Dong, F. Hao et al., Assembling ultrasmall copper-doped ruthenium oxide nanocrystals into hollow porous polyhedra: highly robust electrocatalysts for oxygen evolution in acidic media. Adv. Mater., e1801351 (2018). https://doi.org/10.1002/adma.201801351

[S26] J. Xu, Z. Lian, B. Wei, Y. Li, O. Bondarchuk et al., Strong electronic coupling between ultrafine iridium–ruthenium nanoclusters and conductive, acid-stable tellurium nanoparticle support for efficient and durable oxygen evolution in acidic and neutral media. ACS Catal. **10**, 3571–3579 (2020). https://doi.org/10.1021/acscatal.9b05611

[S27] L. Fu, J. Zhou, L. Zhou, J. Yang, Z. Liu et al., Facile fabrication of exsolved nanoparticle-decorated hollow ferrite fibers as active electrocatalyst for oxygen evolution reaction. Chem. Eng. J. **418**, 129422 (2021). https://doi.org/10.1016/j.cej.2021.129422

[S28] L. Cao, Q. Luo, J. Chen, L. Wang, Y. Lin et al., Dynamic oxygen adsorption on single-atomic Ruthenium catalyst with high performance for acidic oxygen evolution reaction. Nat. Commun. **10**, 4849 (2019). https://doi.org/10.1038/s41467-019-12886-z

[S29] Q. Feng, Q. Wang, Z. Zhang, Y. Xiong, H. Li et al., Highly active and stable ruthenate pyrochlore for enhanced oxygen evolution reaction in acidic medium electrolysis. Appl. Catal. B Environ. **244**, 494–501 (2019). https://doi.org/10.1016/j.apcatb.2018.11.071

[S30] R. Qin, P. Wang, Z. Li, J. Zhu, F. Cao et al., Ru-incorporated nickel diselenide nanosheet arrays with accelerated adsorption kinetics toward overall water splitting. Small **18**, e2105305 (2022). https://doi.org/10.1002/smll.202105305

[S31] H.-J. Liu, C.-Y. Chiang, Y.-S. Wu, L.-R. Lin, Y.-C. Ye et al., Breaking the relation between activity and stability of the oxygen-evolution reaction by highly doping Ru in wide-band-gap $SrTiO_3$ as electrocatalyst. ACS Catal. **12**, 6132–6142 (2022). https://doi.org/10.1021/acscatal.1c05539

[S32] X. Ping, Y. Liu, S. Chen, N. Ran, L. Zheng et al., Tailoring B-site of lead-ruthenate pyrochlore for boosting acidic water oxidation activity and stability. Appl. Catal. B Environ. **318**, 121884 (2022). https://doi.org/10.1016/j.apcatb.2022.121884

[S33] A.N. Singh, M.H. Kim, A. Meena, T.U. Wi, H.W. Lee et al., Na/Al codoped layered cathode with defects as bifunctional electrocatalyst for high-performance Li-ion battery and oxygen evolution reaction. Small **17**, e2005605 (2021). https://doi.org/10.1002/smll.202005605

[S34] K. Huang, C. Lin, G. Yu, P. Du, X. Xie et al., Ru/Se-RuO_2 composites via controlled selenization strategy for enhanced acidic oxygen evolution. Adv. Funct. Mater. **33**, 2211102 (2023). https://doi.org/10.1002/adfm.202211102

[S35] X. Mu, X. Gu, S. Dai, J. Chen, Y. Cui et al., Breaking the symmetry of single-atom catalysts enables an extremely low energy barrier and high stability for large-current-density water splitting. Energy Environ. Sci. **15**, 4048–4057 (2022). https://doi.org/10.1039/d2ee01337a

[S36] Z. Peng, C. Han, C. Huang, Z. Dong, X. Ma Preventing surface passivation of transition metal nanoparticles in oxygen electrocatalyst to extend the lifespan of Zn-air battery. J. Mater. Sci. Technol. **128**, 205–212 (2022). https://doi.org/10.1016/j.jmst.2022.03.033

[S37] M. Kim, H. Ju, J. Kim Single crystalline $Bi_2Ru_2O_7$ pyrochlore oxide nanoparticles as efficient bifunctional oxygen electrocatalyst for hybrid Na-air batteries. Chem. Eng. J. **358**, 11–19 (2019). https://doi.org/10.1016/j.cej.2018.09.204

[S38] L. Qiu, G. Zheng, Y. He, L. Lei, X. Zhang Ultra-small Sn-RuO_2 nanoparticles supported on N-doped carbon polyhedra for highly active and durable oxygen evolution reaction in acidic media. Chem. Eng. J. **409**, 128155 (2021). https://doi.org/10.1016/j.cej.2020.128155

[S39] T. Liu, H. Guo, Y. Chen, Z. Zhang, F. Wang Role of MoO_x surficial modification in enhancing the OER performance of Ru-pyrochlore. Small **19**, e2206698 (2023). https://doi.org/10.1002/smll.202206698

[S40] X. Duan, P. Li, D. Zhou, S. Wang, H. Liu et al., Stabilizing single-atomic ruthenium by ferrous ion doped NiFe-LDH towards highly efficient and sustained water oxidation. Chem. Eng. J. **446**, 136962 (2022). https://doi.org/10.1016/j.cej.2022.136962

[S41] G. Chen, T. Wang, J. Zhang, P. Liu, H. Sun et al., Accelerated hydrogen evolution kinetics on NiFe-layered double hydroxide electrocatalysts by tailoring water dissociation active sites. Adv. Mater. **30**, 1706279 (2018). https://doi.org/10.1002/adma.201706279

[S42] J. Joo, H. Jin, A. Oh, B. Kim, J. Lee et al., An IrRu alloy nanocactus on $Cu_{2-x}S$@IrS_y as a highly efficient bifunctional electrocatalyst toward overall water splitting in acidic electrolytes. J. Mater. Chem. A **6**, 16130–16138 (2018). https://doi.org/10.1039/c8ta04886j

[S43] Z.L. Zhao, Q. Wang, X. Huang, Q. Feng, S. Gu et al., Boosting the oxygen evolution reaction using defect-rich ultra-thin ruthenium oxide nanosheets in acidic media. Energy Environ. Sci. **13**, 5143–5151 (2020). https://doi.org/10.1039/d0ee01960g

[S44] T. Feng, G. Yu, S. Tao, S. Zhu, R. Ku et al., A highly efficient overall water splitting ruthenium-cobalt alloy electrocatalyst across a wide pH range *via* electronic coupling with carbon dots. J. Mater. Chem. A **8**, 9638–9645 (2020). https://doi.org/10.1039/D0TA02496A

[S45] B. Tang, X. Yang, Z. Kang, L. Feng Crystallized $RuTe_2$ as unexpected bifunctional catalyst for overall water splitting. Appl. Catal. B Environ. **278**, 119281 (2020). https://doi.org/10.1016/j.apcatb.2020.119281

[S46] J. He, W. Li, P. Xu, J. Sun Tuning electron correlations of RuO_2 by co-doping of Mo and Ce for boosting electrocatalytic water oxidation in acidic media. Appl. Catal. B Environ. **298**, 120528 (2021). https://doi.org/10.1016/j.apcatb.2021.120528

[S47] Y. Zhang, D. Wang, C. Ye, F. Gao, Z. Li et al., Regulation of crystallinity and defects on CoNiRuOx nanocages for enhanced oxygen evolution reaction. Chem. Eng. J. **466**, 143059 (2023). https://doi.org/10.1016/j.cej.2023.143059

[S48] Y. Zhao, M. Xi, Y. Qi, X. Sheng, P. Tian et al., Redirecting dynamic structural evolution of nickel-contained RuO_2 catalyst during electrochemical oxygen evolution reaction. J. Energy Chem. **69**, 330–337 (2022). https://doi.org/10.1016/j.jechem.2022.01.030

[S49] H. Wang, P. Yang, X. Sun, W. Xiao, X. Wang et al., Co-Ru alloy nanoparticles decorated onto two-dimensional nitrogen doped carbon nanosheets towards hydrogen/oxygen evolution reaction and oxygen reduction reaction. J. Energy Chem. **87**, 286–294 (2023). https://doi.org/10.1016/j.jechem.2023.08.039

[S50] H. Xu, T. Liu, S. Bai, L. Li, Y. Zhu et al., Cation exchange strategy to single-atom noble-metal doped CuO nanowire arrays with ultralow overpotential for H_2O splitting. Nano Lett. **20**, 5482–5489 (2020). https://doi.org/10.1021/acs.nanolett.0c02007

[S51] C. Hu, L. Zhang, Z.-J. Zhao, A. Li, X. Chang et al., Synergism of geometric construction and electronic regulation: 3D Se-(NiCo)S_x/$(OH)_x$ nanosheets for highly efficient overall water splitting. Adv. Mater. **30**, e1705538 (2018). https://doi.org/10.1002/adma.201705538

[S52] N. Liu, K. Yin, C. Si, T. Kou, Y. Zhang et al., Hierarchically porous nickel–iridium–ruthenium–aluminum alloys with tunable compositions and electrocatalytic activities towards the oxygen/hydrogen evolution reaction in acid electrolyte. J. Mater. Chem. A **8**, 6245–6255 (2020). https://doi.org/10.1039/D0TA00445F

[S53] M. Liu, S. Liu, Q. Mao, S. Yin, Z. Wang et al., Ultrafine ruthenium–iridium–tellurium nanotubes for boosting overall water splitting in acidic media. J. Mater. Chem. A **10**, 2021–2026 (2022). https://doi.org/10.1039/d1ta07789a

[S54] S. Chen, C. Wang, F. Gao, Y. Yang, M. Huang et al., An indium-induced-synthesis $In_{0.17}Ru_{0.83}O_2$ nanoribbon as highly active electrocatalyst for oxygen evolution in acidic media at high current densities above 400 mA cm^{-2}. J. Mater. Chem. A **10**, 3722–3731 (2022). https://doi.org/10.1039/d1ta10022j

[S55] Y. Wang, Q. Sun, Z. Wang, W. Xiao, Y. Fu et al., *In situ* phase-reconfiguration to synthesize Ru, B Co-doped nickel phosphide for energy-efficient hydrogen generation in alkaline electrolytes. J. Mater. Chem. A **10**, 16236–16242 (2022). https://doi.org/10.1039/D2TA02685F

[S56] Z. Jin, J. Lyu, K. Hu, Z. Chen, G. Xie et al., Eight-component nanoporous high-entropy oxides with low Ru contents as high-performance bifunctional catalysts in Zn-air batteries. Small **18**, e2107207 (2022). https://doi.org/10.1002/smll.202107207

[S57] W. Jiang, J. Wang, Y. Jiang, Y. Wu, B. Liu et al., Multivalent ruthenium immobilized by self-supported NiFe–organic frameworks for efficient electrocatalytic overall water splitting. J. Mater. Chem. A **11**, 2769–2779 (2023). https://doi.org/10.1039/d2ta06560f

[S58] K. Du, L. Zhang, J. Shan, J. Guo, J. Mao et al., Interface engineering breaks both stability and activity limits of RuO_2 for sustainable water oxidation. Nat. Commun. **13**, 5448 (2022). https://doi.org/10.1038/s41467-022-33150-x
